# Supplementary material for: MemPrep, a new technology for isolating organellar membranes provides fingerprints of lipid bilayer stress
Source: EMBO J. 2024 Mar 15;43(8):13. doi: 10.1038/s44318-024-00063-y (PMC11021466; doi:10.1038/s44318-024-00063-y)
Supplement: Supplementary file 1 — Appendix [file 44318_2024_63_MOESM1_ESM.pdf]

APPENDIX

A new technology for isolating organellar membranes provides fingerprints of lipid bilayer stress

Authors: John Reinhard, Leonhard Starke, Christian Klose, Per Haberkant, Henrik Hammarén, Frank Stein, Ofir Klein, Charlotte Berhorst, Heike Stumpf, James P. Sáenz, Jochen Hub, Maya Schuldiner, Robert Ernst

Correspondence to: robert.ernst@uks.eu

Table of contents

1. Appendix Figure S1 page 2
2. Appendix Figure S2 page 4
3. Appendix Figure S3 page 5
4. Appendix Figure S4 page 6
5. Appendix Figure S5 page 8
6. Appendix Figure S6 page 9
7. Appendix Supplementary Methods page 10

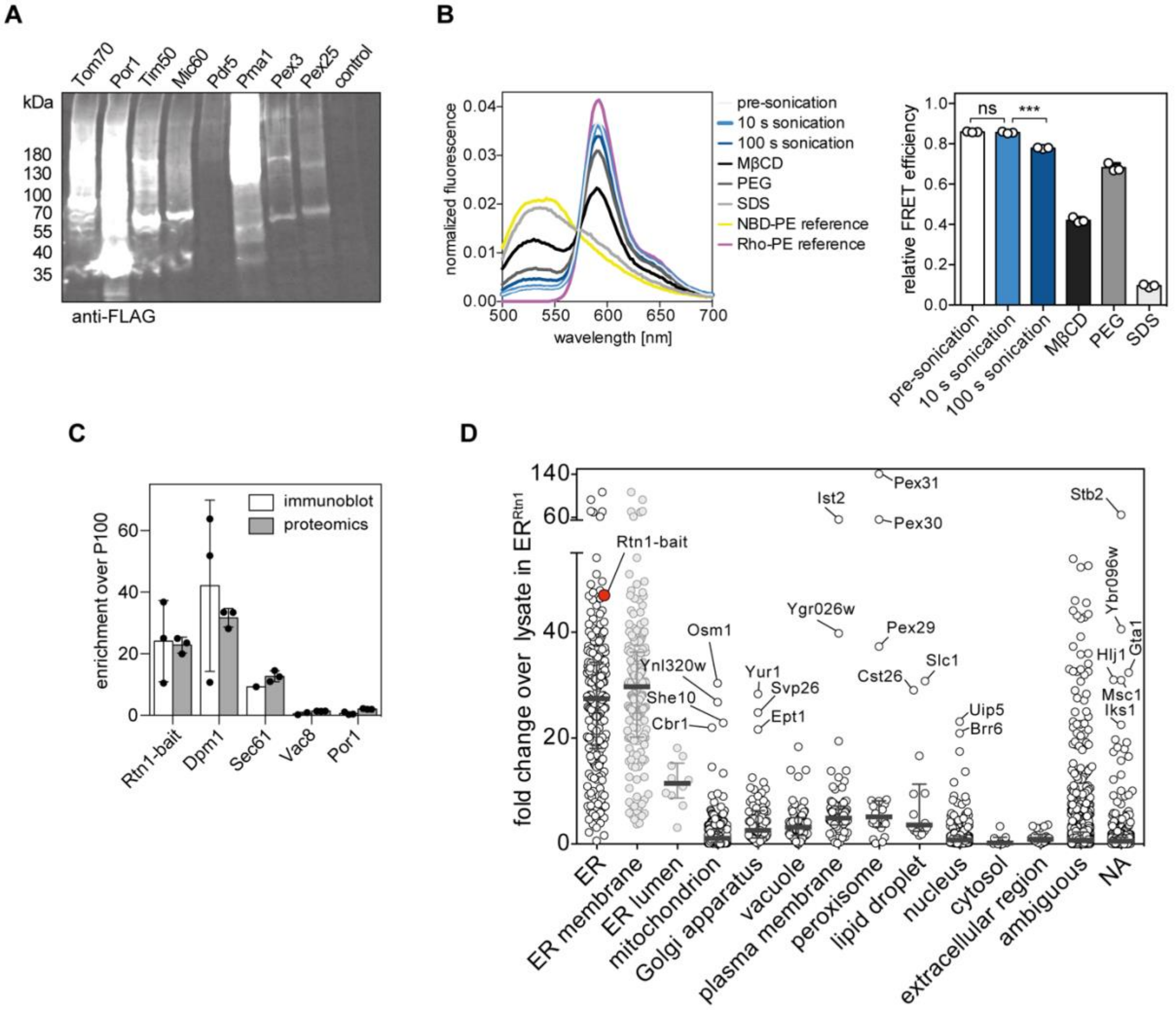

Appendix Figure S1. Analytical data from MemPrep-derived samples has predictive power. (A) From a systematic collection of strains in which every protein is tagged with a C-terminal bait tag (myc-3C-3xFLAG) we generated cell lysates for exemplary strains embedded in different organellar membranes. **(B)** Fluorescence spectra and calculation of relative FRET efficiencies in mixtures of labeled liposomes and a ~15.4-fold excess (based on membrane phospholipid content) of unlabeled P100 microsomes after sonication as performed during MemPrep procedure (10 s sonication), after extensive sonication (100 s sonication), or upon incubation with 18 mM methyl beta cyclodextrin (MβCD), 40% (w/v) polyethylene glycol 8000 (PEG 8000), or 1% sodium dodecyl sulfate (SDS). A low relative FRET efficiency is the result of decreased average proximity of the two FRET-pair fluorophores and indicative for either fusion of labeled liposomes with unlabeled P100 microsomes or lipid exchange between vesicles. Data are presented as individual data points and mean ± SD (n = 3 independent experiments). Statistical significance was tested using an unpaired parametric t test with Welch's correction. ns: not significant; ***$p \leq 0.001$. **(C)** Correlation of mean enrichments of organellar markers in ER membranes derived by MemPrep over P100 microsomes determined by either immunoblot analysis or proteomics. Immuno-isolation bait protein (myc-Rtn1), ER markers Dpm1 and Sec61, vacuole marker Vac8, and outer mitochondrial membrane marker Por1. Error bars indicate SD (n = 3 for Rtn1-bait, Dpm1, and Por1 immunoblot; n = 1 for Sec61 immunoblot; n = 2 for Vac8 immunoblot; n = 3 for all markers in proteomics data). **(D)** Mean enrichment (fold change over lysate) of each individual identified protein in our untargeted TMT-labeling proteomics data (Figure 1E, n = 3). The bait protein 'Rtn1-bait' via which MemPrep was perfomed is indicated. Several proteins that were highly enriched in our ER membrane preparation via the Rtn1-bait are uniquely annotated to other organelles. Annotations of subcellular location were retrieved from UniProt (accessed 27.01.2023). A subsequent consultation of the *Saccharomyces* Genome Database gene description and GOterms (accessed 03.01.2024)

reveals that many of those highly enriched proteins annotated to other organelles were previously observed in the ER or the nuclear envelope (Ist2, Pex30, Pex29, Brr6, She10, YBR096W, YEL043W, Hlj1, YGR026W), or feature a dual localization to the ER and other organelles (Osm1, Slc1, Svp26, Yur1, Ept1, Pex31, Msc1). 'NA' stands for no annotation for subcellular location.
Source data for this figure are available online.

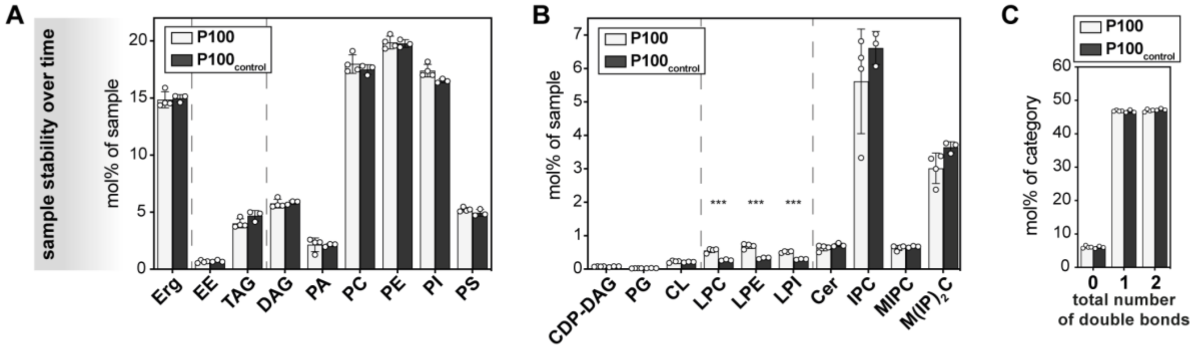

Appendix Figure S2. Lysolipids are depleted from the samples during the isolation procedure. To control stability of the sample an aliquot of P100 microsomes was incubated at 4 °C and overhead rotation ($P100_{control}$) while the remaining sample was purified by immuno-isolation. **(A)** Abundance of detected lipid classes in microsomes (P100) and control microsomes after incubation for 8 h at 4 °C ($P100_{control}$). **(B)** Lipid class distribution showing significantly less lyso-phospholipids in control microsomes ($P100_{control}$). **(C)** The total number of double bonds in membrane glycerolipids is not changed.

In (A-C), data are presented as individual data points and mean ± SD (P100, n = 4 biological replicates; $P100_{control}$, n = 3 biological replicates). Statistical significance was tested by multiple t tests correcting for multiple comparisons using the method of Benjamini, Krieger and Yekutieli, with Q = 1%, without assuming consistent standard deviations. ***$p \leq 0.001$. Source data for this figure are available online.

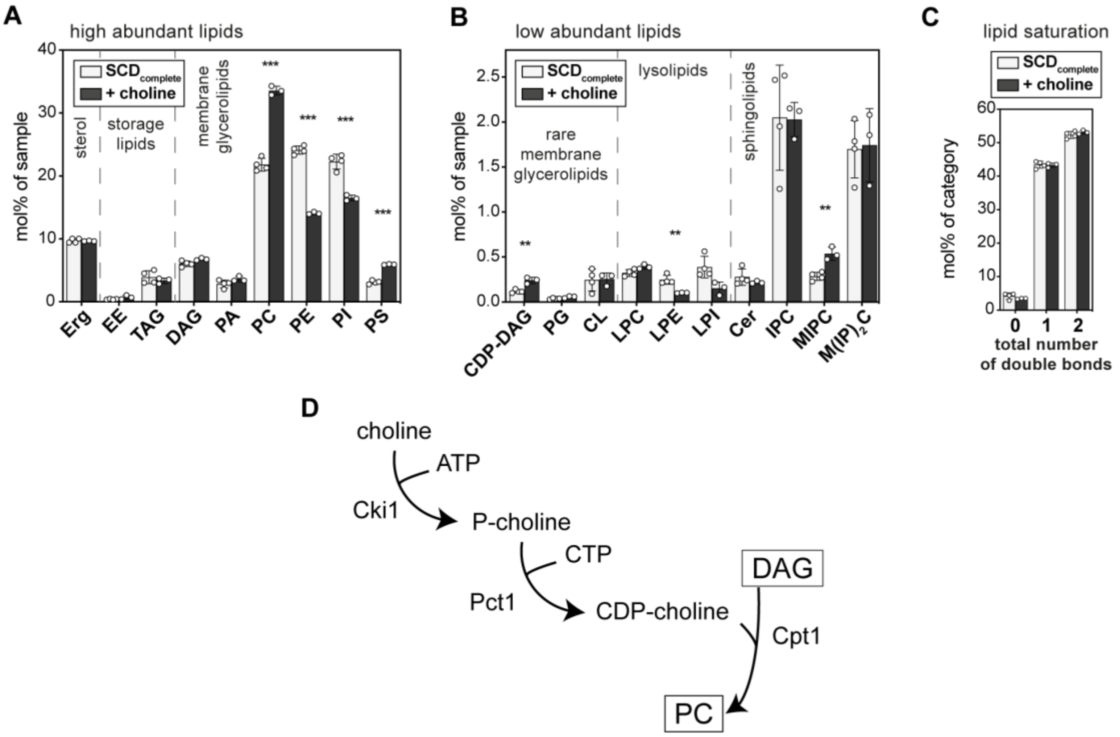

Appendix Figure S3. Metabolic interference with choline induces dramatic lipidome changes without activation of the UPR. $SCD_{complete}$ medium containing 2 mM choline (+ choline) was inoculated with Rtn1-bait cells to an OD_{600} of 0.1 from an overnight pre-culture and cells were harvested at an OD_{600} of 1.0. ER derived membranes were purified by differential centrifugation and immuno-isolation and subsequently analyzed by quantitative shotgun lipidomics. **(A)** Lipid class composition given as mol% of all lipids in the sample. **(B)** Less abundant classes. **(C)** Total number of double bonds in membrane glycerolipids (except CL which has four acyl chains) as mol% of this category. Lipid data for $SCD_{complete}$ in panel A-C are identical with the data presented in Figure 2A-C (ER^{Rtn1}). **(D)** Lipid metabolic map of PC biosynthesis from external choline sources.

In (A-C), data are presented as individual data points and mean ± SD ($SCD_{complete}$, n = 4 biological replicates; + choline, n = 3 biological replicates). Statistical significance was tested by multiple t tests correcting for multiple comparisons using the method of Benjamini, Krieger and Yekutieli, with Q = 1%, without assuming consistent standard deviations. $^{**}p \leq 0.01$, $^{***}p \leq 0.001$. Source data for this figure are available online.

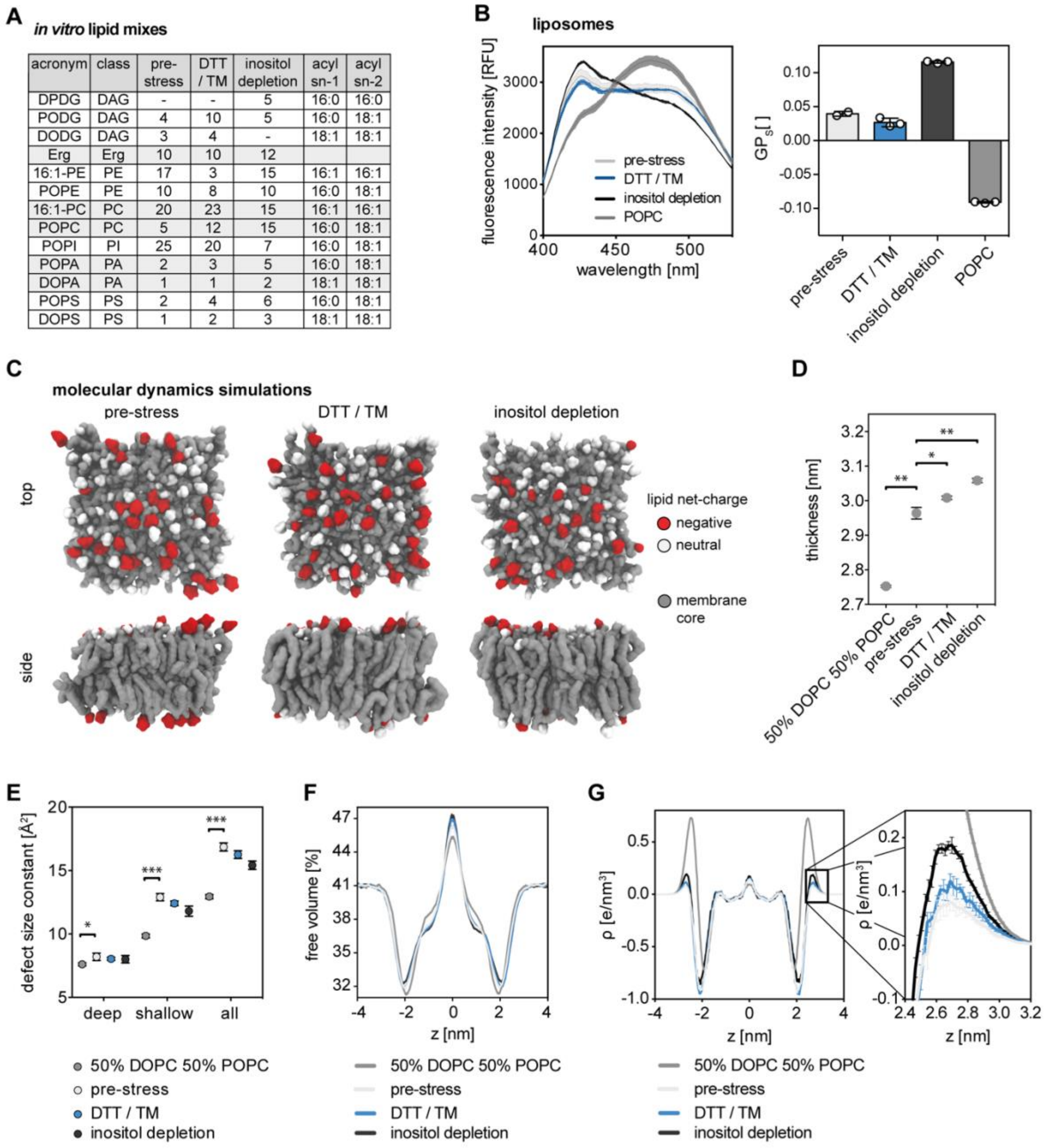

A *in vitro* lipid mixes

acronym	class	pre-stress	DTT / TM	inositol depletion	acyl sn-1	acyl sn-2
DPDG	DAG	-	-	5	16:0	16:0
PODG	DAG	4	10	5	16:0	18:1
DODG	DAG	3	4	-	18:1	18:1
Erg	Erg	10	10	12		
16:1-PE	PE	17	3	15	16:1	16:1
POPE	PE	10	8	10	16:0	18:1
16:1-PC	PC	20	23	15	16:1	16:1
POPC	PC	5	12	15	16:0	18:1
POPI	PI	25	20	7	16:0	18:1
POPA	PA	2	3	5	16:0	18:1
DOPA	PA	1	1	2	18:1	18:1
POPS	PS	2	4	6	16:0	18:1
DOPS	PS	1	2	3	18:1	18:1

Appendix Figure S4. An *in vitro* lipid mixture that mimics the ER membrane under prolonged proteotoxic and lipid bilayer stresses. (A) Based on our ER membrane lipidomics data we propose commercially available lipid mixtures that mimic the ER under non-stress (pre-stress), prolonged proteotoxic stress (DTT / TM) or lipid bilayer stress conditions (inositol depletion). **(B)** Unilamellar liposomes were prepared by rehydrating dried lipids and extrusion. General polarization (GP) of fluorescence spectra using the membrane probe C-laurdan ranging from +1 (most ordered membrane) to -1 (most disordered) indicate that the proposed lipid mixtures have distinct physical properties. Data are presented as individual data points and mean ± SD (n = 3 independent liposome preparations). **(C)** Molecular dynamics (MD) simulations of proposed commercially available *in vitro* ER membrane lipid mixes for non-stress ER (pre-stress) and ER under two different lipid bilayer stress conditions (DTT / TM, inositol depletion). Snapshots were taken after 100 ns. **(D)** Thickness measurements taken from MD simulations. Data are presented as mean ± SEM (n = 3). Statistical significance was tested using an unpaired parametric t test with Welch's correction. *p ≤ 0.05, **p ≤ 0.01. **(E)** Determination of defect size constants in MD simulation of model membranes. Data are presented as mean ± SEM (n = 3). Statistical significance was tested using an unpaired parametric t test with Welch's correction. *p ≤ 0.05, ***p ≤ 0.001. **(F)** Free volume calculations from MD simulations. Data are presented as mean ± SEM (n

= 3). The SEM is within the range of the thickness of the plotted line. **(G)** Charge distribution computed from partial charge density during MD simulations. Data are presented as mean ± SEM (n = 3). Source data for this figure are available online.

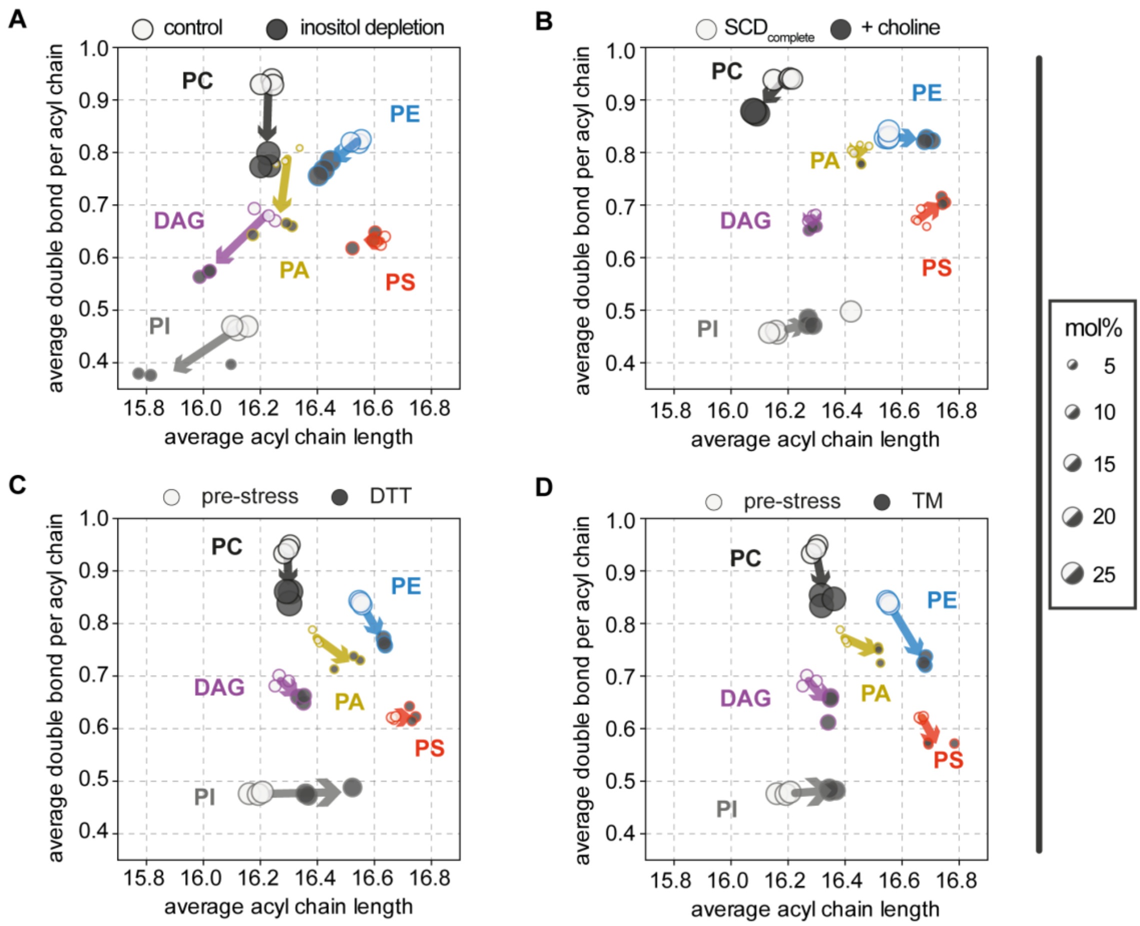

Appendix Figure S5. Changes of average acyl chain length and unsaturation in abundant glycerolipid classes under conditions of ER stress. Arrow diagrams showing changes in acyl chain composition (average number of double bonds per acyl chain, average length per acyl chain) for each glycerolipid class in our ER lipidome data (n = 3 biological replicates for all conditions except ‘$\text{SCD}_{\text{complete}}$’, where n = 4). **(A)** Acyl chain changes upon lipid bilayer stress by inositol depletion (based on data from Figure 3B, C). Lipid acyl chains from all measured classes tend to get shorter and more saturated. **(B)** Acyl chain changes upon addition of 2 mM choline (based on data from Appendix Figure S3A, B). Acyl chains of PC lipids tend to get shorter and more saturated. Acyl chains of PE, PS and PI lipids tend to get longer. **(C)** Acyl chain changes after prolonged proteotoxic stress by DTT (based on data from Figure 4D, E). **(D)** Acyl chain changes after prolonged proteotoxic stress by TM (based on data from Figure 4D, E). Similar changes are measured for both proteotoxic stress inducers, DTT and TM. PC acyl chains get more saturated, PI acyl chains get longer. Acyl chains in the other lipid classes tend to become longer and more saturated. Circle diameters are proportional to abundance of the respective lipid class in the ER membrane of indicated growth condition. Source data for this figure are available online.

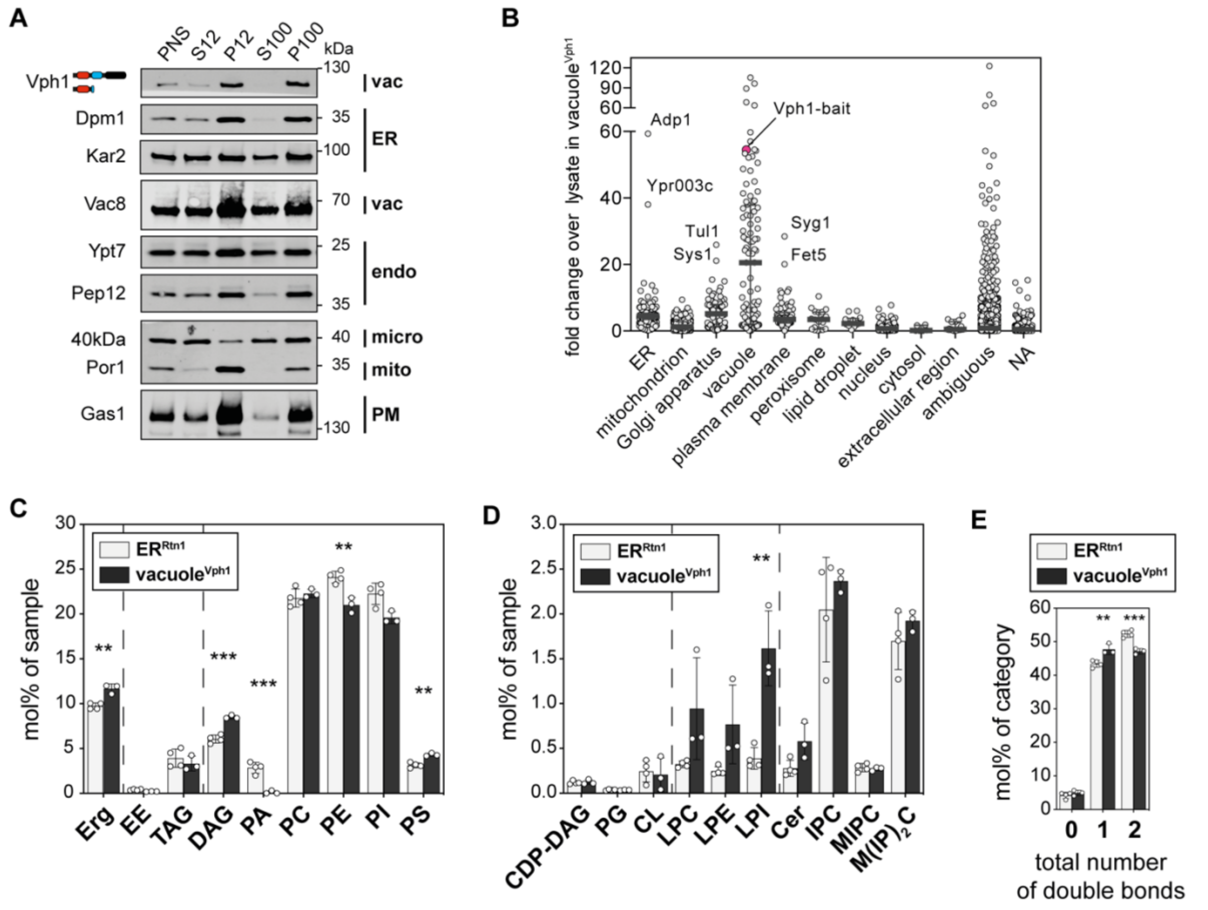

Appendix Figure S6. Lipidomics of the vacuole. SCD$_{complete}$ medium was inoculated with Vph1-bait cells to an OD_{600} of 0.1 from an overnight pre-culture and cells were harvested at an OD_{600} of 1.0. Membranes of the vacuole were purified by MemPrep and subsequently analyzed by TMT-labeling proteomics and quantitative shotgun lipidomics. **(A)** Differential centrifugation yields a crude membrane fraction (P100) that is subsequently used as input for immuno-isolation. **(B)** Mean enrichment (fold change over lysate) of each individual identified protein in our untargeted TMT-labeling proteomics data (Figure 6B, n = 3). The bait protein 'Vph1-bait' via which MemPrep was performed is indicated. Our vacuole membrane preparation seemed to contain several enriched proteins with annotations of non-vacuole organelles. Annotations of subcellular location were retrieved from UniProt (accessed 27.01.2023). A subsequent consultation of the *Saccharomyces* Genome Database gene description and GOterms (accessed 03.01.2024) reveals that many of those highly enriched proteins annotated to other organelles were previously observed in the vacuole or feature a dual localization to the vacuole and other organelles (Tul1, Syg1, Fet5, Adp1). **(C)** Lipid class composition given as mol% of all lipids in the sample. **(D)** Less abundant classes. **(E)** Total number of double bonds in membrane glycerolipids (except CL which has four acyl chains) as mol% of this category.

In (C-E), lipid data for ER^{Rtn1} are identical with the data presented in Figure 2A-C. Lipid data for vacuole^{Vph1} in panel C and D are identical with the data presented in Figure 6C and D. Data are presented as individual data points and mean ± SD (n = 3 biological replicates). Statistical significance was tested by multiple t tests correcting for multiple comparisons using the method of Benjamini, Krieger and Yekutieli, with Q = 1%, without assuming consistent standard deviations. $^{**}p \le 0.01$, $^{***}p \le 0.001$. Source data for this figure are available online.

Appendix Supplementary Methods

Bait-tagging cassette on plasmid pRE866 coding regions:

myc-HRV 3C site-3xFLAG (amino acids)
GGGGGGEQKLISEEDLGSGLEVLFQGPGSGDYKDHDGDYKDHDIDYKDDDDK*

KanR (aminoglycoside phosphotransferase) (amino acids)
MGKEKTHVSRPRLNSNMDADLYGYKWARDNVGQSGATIYRLYGKPDAPELFLKHGKGSVA
NDVTDEMVRLNWLTEFMPLPTIKHFIRTPDDAWLLTTAIPGKTAFQVLEEYPDSGENIVDALA
VFLRRLHSIPVCNCPFNSDRVFRLAQAQSRMNNGLVDASDFDDERNGWPVEQVWKEMHK
LLPFSPDSVVTHGDFSLDNLIFDEGKLIGCIDVGRVGIADRYQDLAILWNCLGEFSPSLQKRL
FQKYGIDNPDMNKLQFHLMLDEFF*

DNA sequence
gggggaggcgggggtggagaacaaaagttgatttctgaagaagatttggggtcaggtctggaagttctgttccaggggcccgga
tctggcgactacaaagaccatgacggtgattataaagatcatgacatcgactacaaggatgacgatgacaagtagggcgcgcc
acttctaaataagcgaatttcttatgatttatgatttttattattaaataagttataaaaaaaataagtgtatacaaattttaaagtgactctt
aggttttaaaacgaaaattcttattcttgagtaactctttcctgtaggtcaggttgctttctcaggtatagtatgaggtcgctcttattgacc
acacctctaccggcagatccgctagggataacagggtaatatagatctgtttagcttgcctcgtccccgccgggtcacccggcca
gcgacatggaggcccagaataccctccttgacagtcttgacgtgcgcagctcaggggcatgatgtgactgtcgcccgtacatttag
cccatacatccccatgtataatcatttgcatccatacattttgatggccgcacggcgcgaagcaaaaattacggctcctcgctgcag
acctgcgagcagggaaacgctcccctcacagacgcgttgaattgtccccacgccgcgcccctgtagagaaatataaaaggtta
ggatttgccactgaggttcttctttcatatacttccttttaaaatcttgctaggatacagttctcacatcacatccgaacataaacaacca
tgggtaaggaaaagactcacgtttcgaggccgcgattaaattccaacatggatgctgatttatatgggtataaatgggctcgcgata
atgtcgggcaatcaggtgcgacaatctatcgattgtatgggaagcccgatgcgccagagttgtttctgaaacatggcaaaggtag
cgttgccaatgatgttacagatgagatggtcagactaaactggctgacggaatttatgcctcttccgaccatcaagcattttatccgta
ctcctgatgatgcatggttactcaccactgcgatccccggcaaaacagcattccaggtattagaagaatatcctgattcaggtgaa
aatattgttgatgcgctggcagtgttcctgcgccggttgcattcgattcctgtttgtaattgtccttttaacagcgatcgcgtatttcgtctcg
ctcaggcgcaatcacgaatgaataacggtttggttgatgcgagtgattttgatgacgagcgtaatggctggcctgttgaacaagtct
ggaaagaaatgcataagcttttgccattctcaccggattcagtcgtcactcatggtgatttctcacttgataaccttatttttgacgaggg
gaaattaataggttgtattgatgttggacgagtcggaatcgcagaccgataccaggatcttgccatcctatggaactgcctcggtga
gttttctccttcattacagaaacggctttttcaaaaatatggtattgataatcctgatatgaataaattgcagtttcatttgatgctcgatga
gtttttctaatcagtactgacaataaaaagattcttgttttcaagaacttgtcatttgtatagtttttttatattgtagttgttctattttaatcaaat
gttagcgtgatttatattttttttcgcctcgacatcatctgcccagatgcgaagttaagtgcgcagaaagtaatatcatgcgtcaatcgt
atgtgaatgctggtcgctatactg
