# Supplementary figures and images for: MemPrep, a new technology for isolating organellar membranes provides fingerprints of lipid bilayer stress
Source: EMBO J. 2024 Mar 15;43(8):13. doi: 10.1038/s44318-024-00063-y (PMC11021466; doi:10.1038/s44318-024-00063-y)

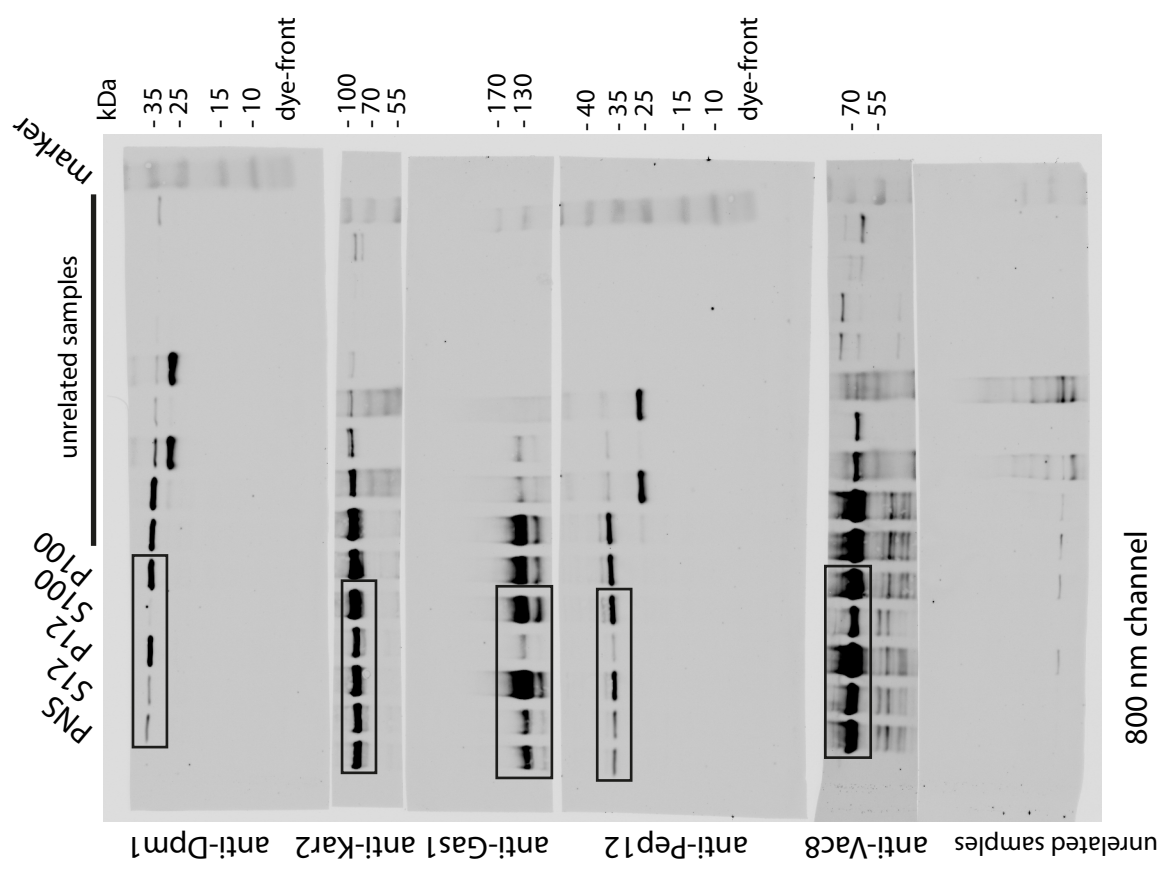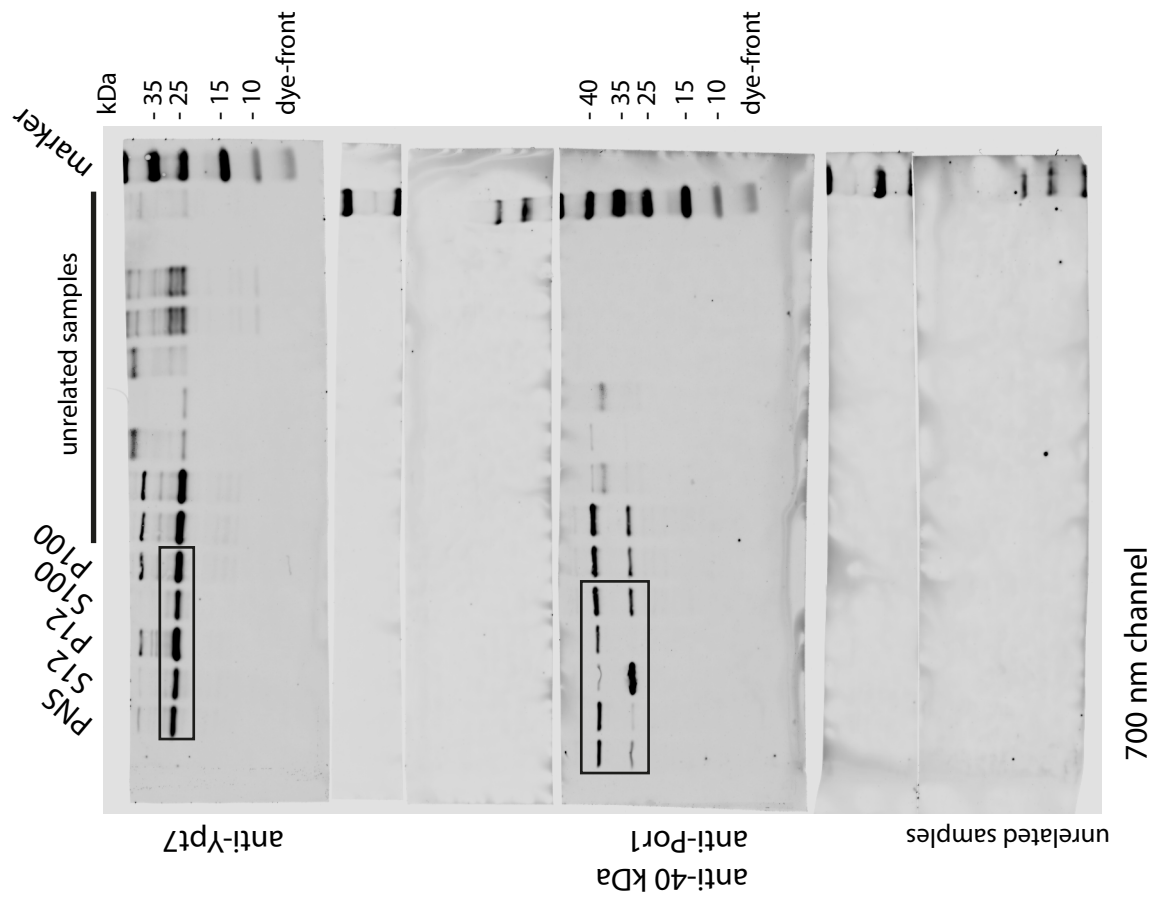

merge

700 nm channel

800 nm channel

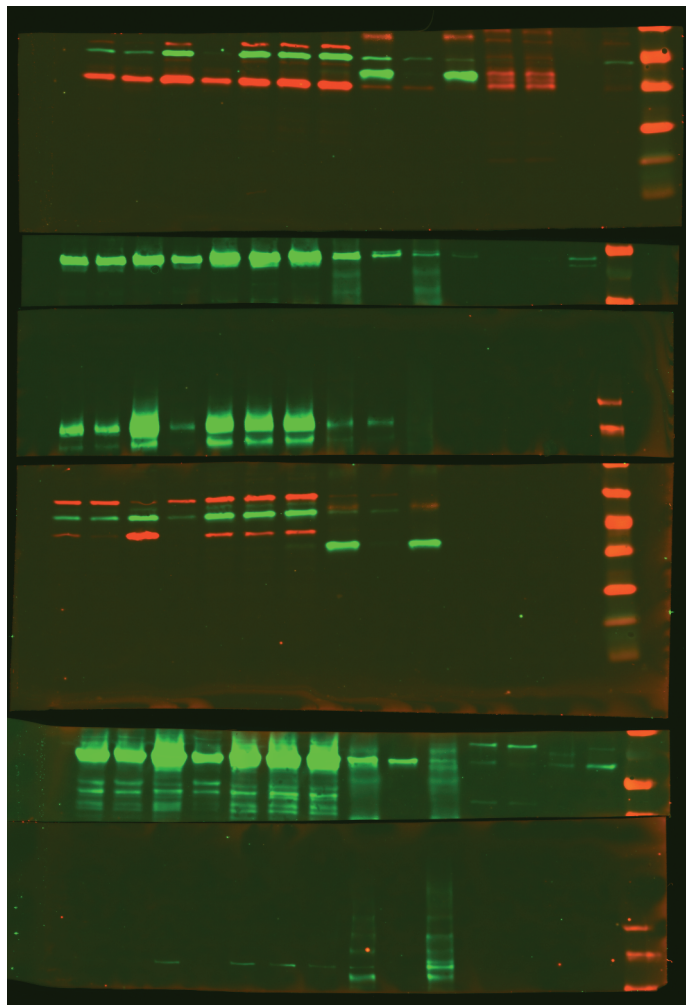

Supplement: Supplementary file 2 — Source Data Fig. 1 [file 44318_2024_63_MOESM2_ESM.zip › Figure 1B_immunoblots.pdf]

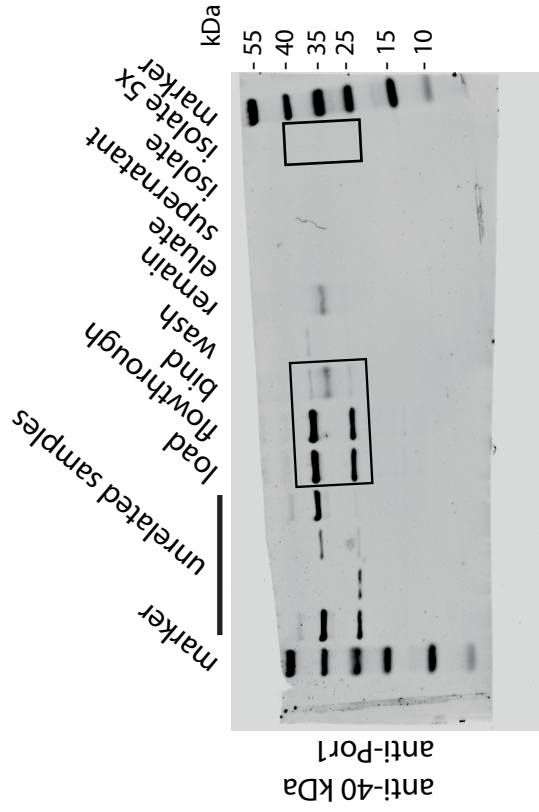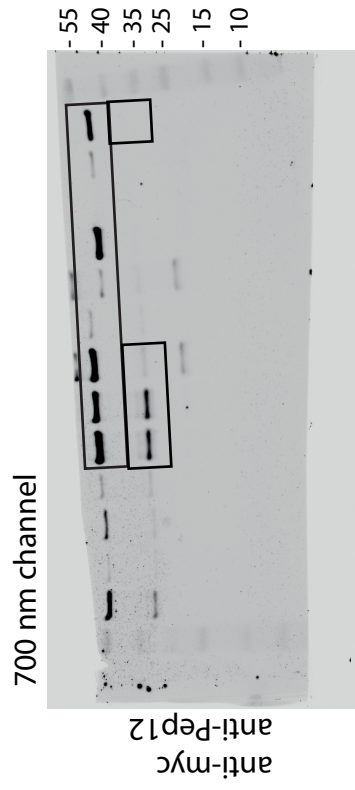

800 nm channel

merge

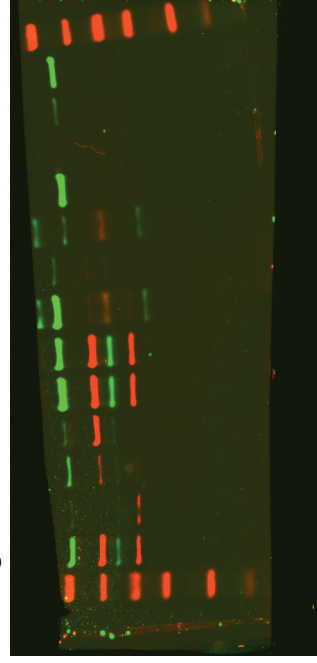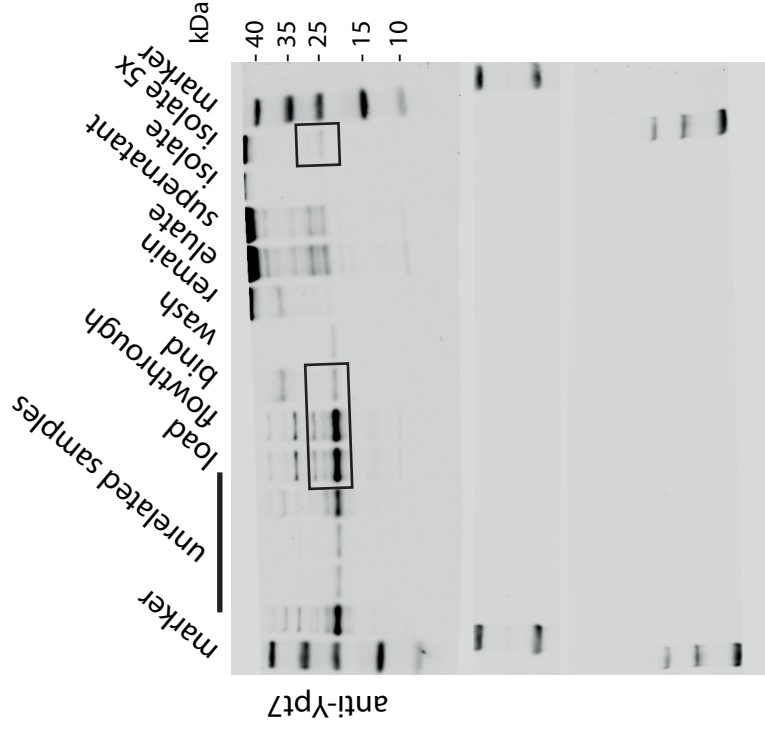

700 nm channel

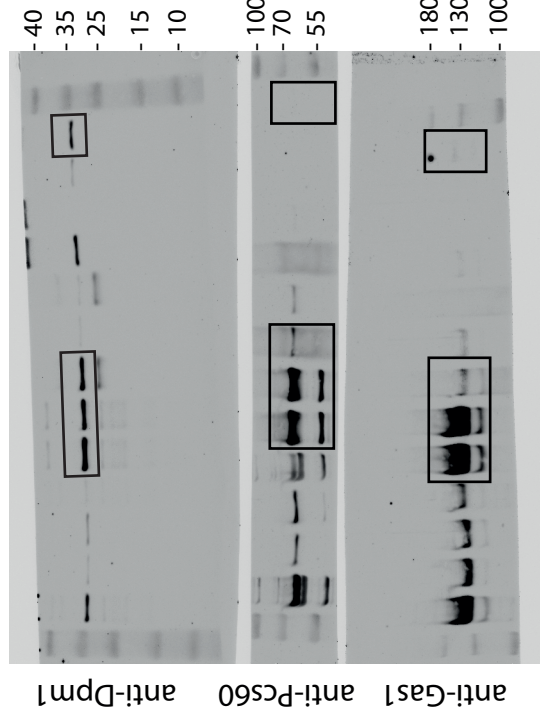

800 nm channel

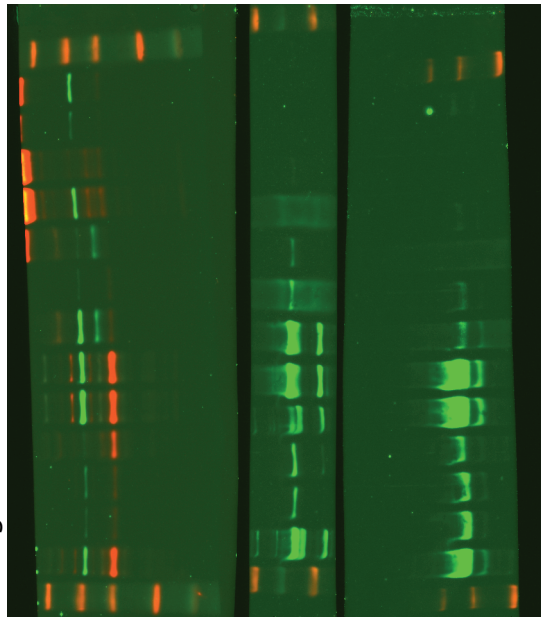

700 nm channel  
800 nm channel

700 nm channel  
800 nm channel

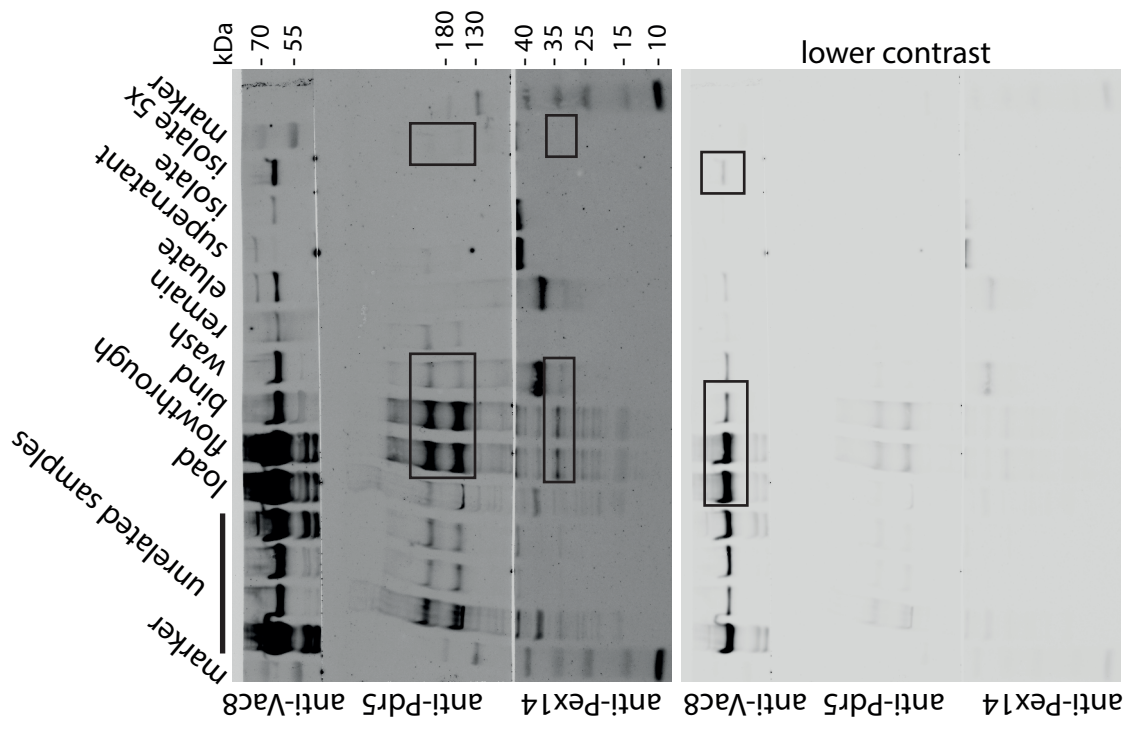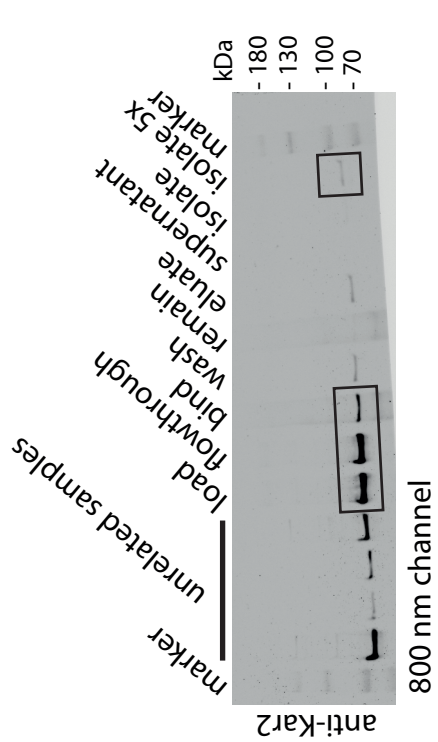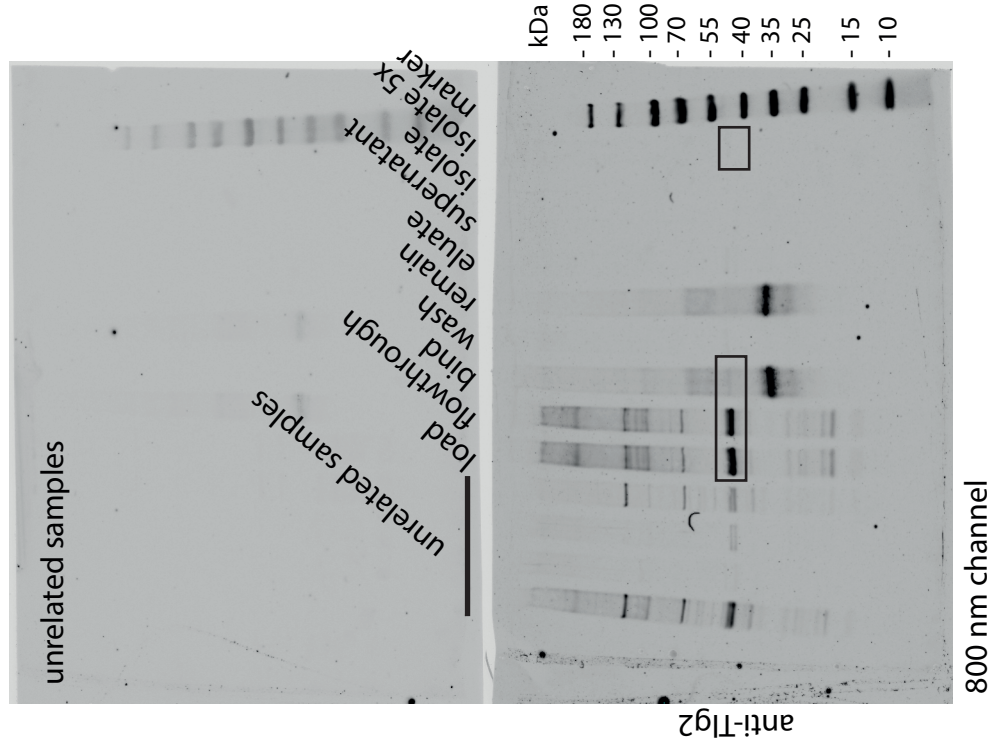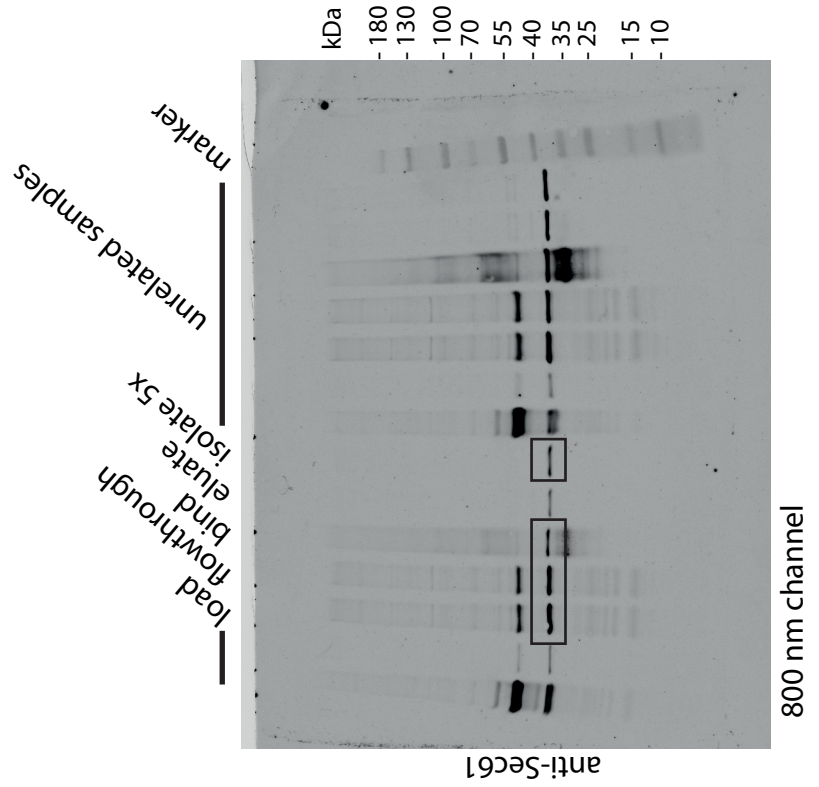

800 nm channel

Supplement: Supplementary file 2 — Source Data Fig. 1 [file 44318_2024_63_MOESM2_ESM.zip › Figure 1D_immunoblots.pdf]

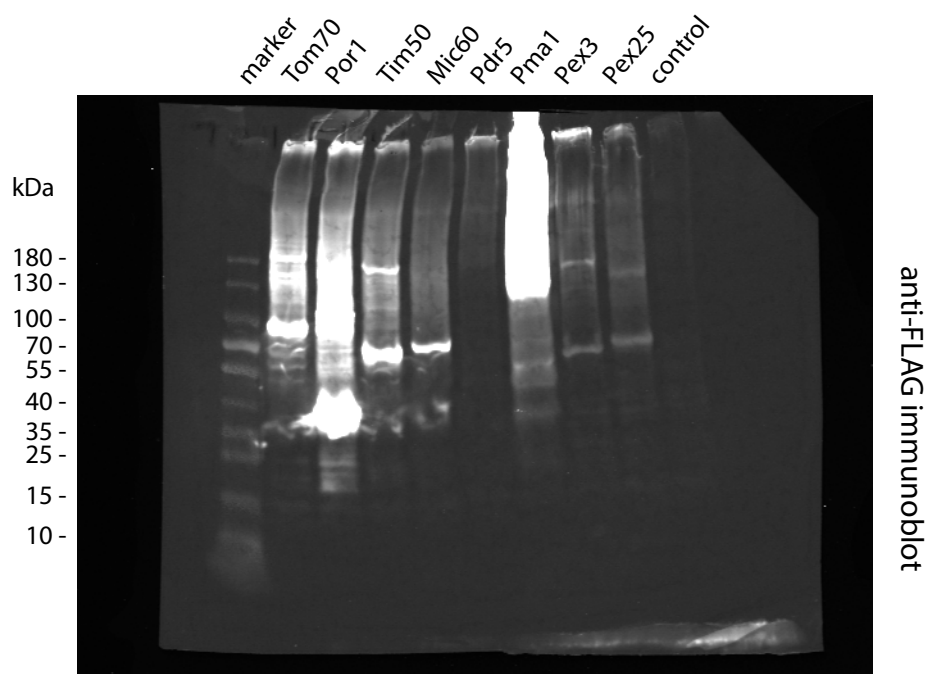

high contrast

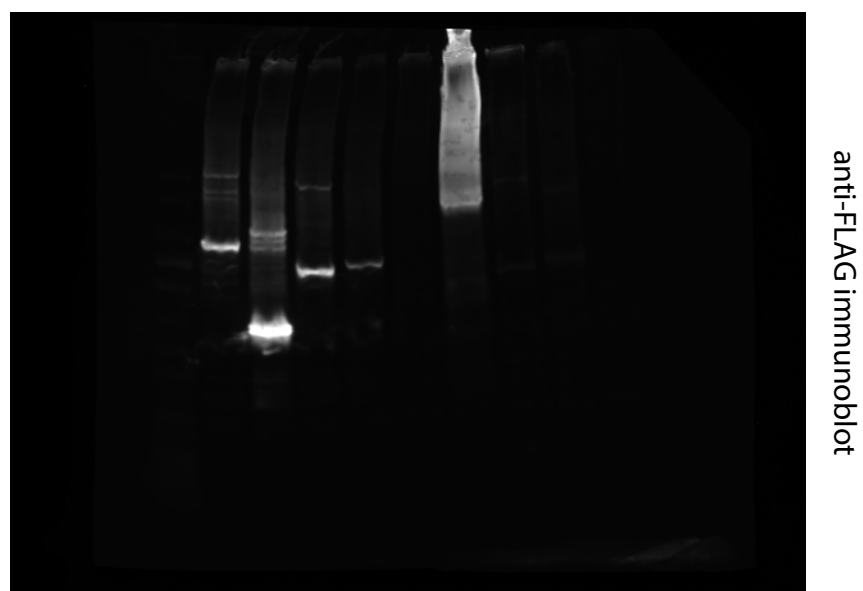

low contrast

Supplement: Supplementary file 8 — EV and Appendix Figure Source Data [file 44318_2024_63_MOESM8_ESM.zip › Appendix Figure S1A_immunoblots.pdf]

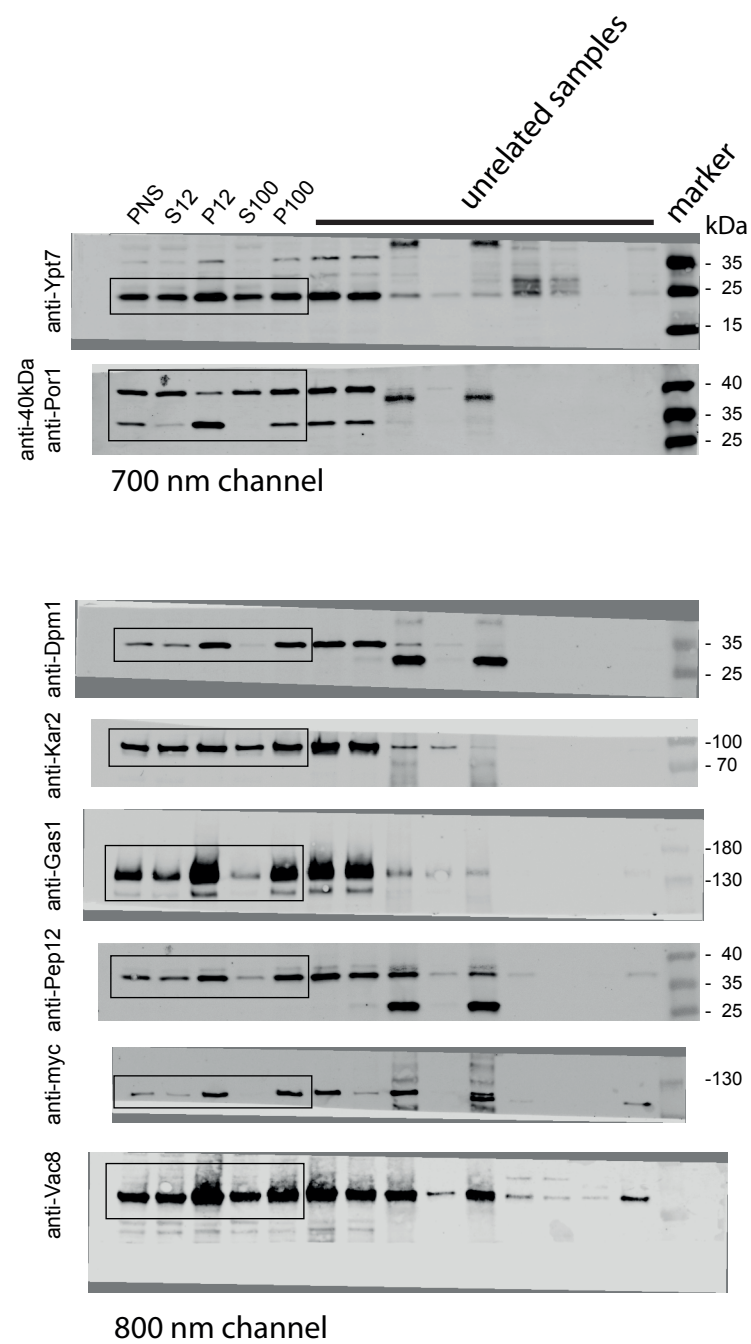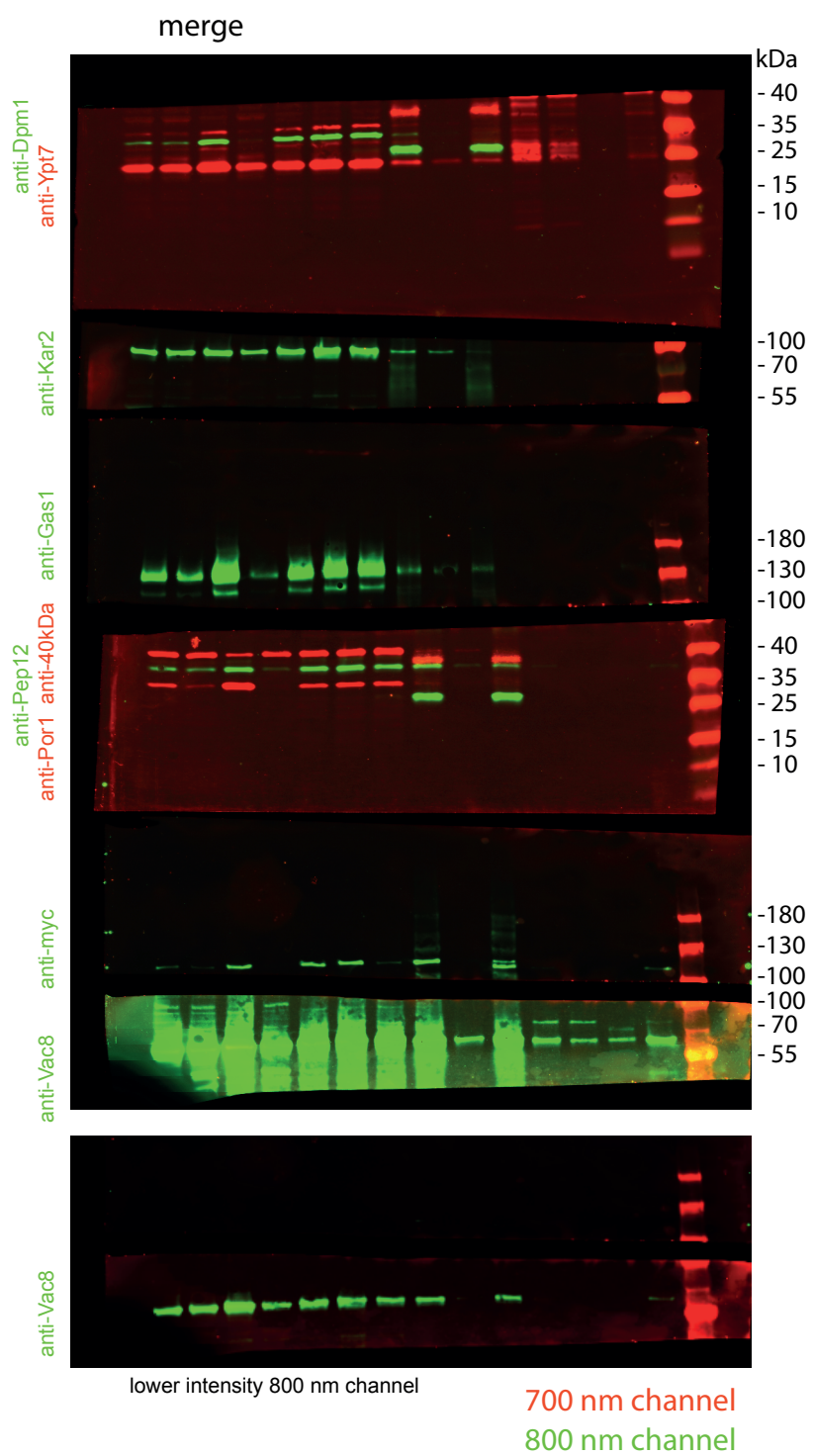

Supplement: Supplementary file 8 — EV and Appendix Figure Source Data [file 44318_2024_63_MOESM8_ESM.zip › Appendix Figure S6_immunoblots.pdf]

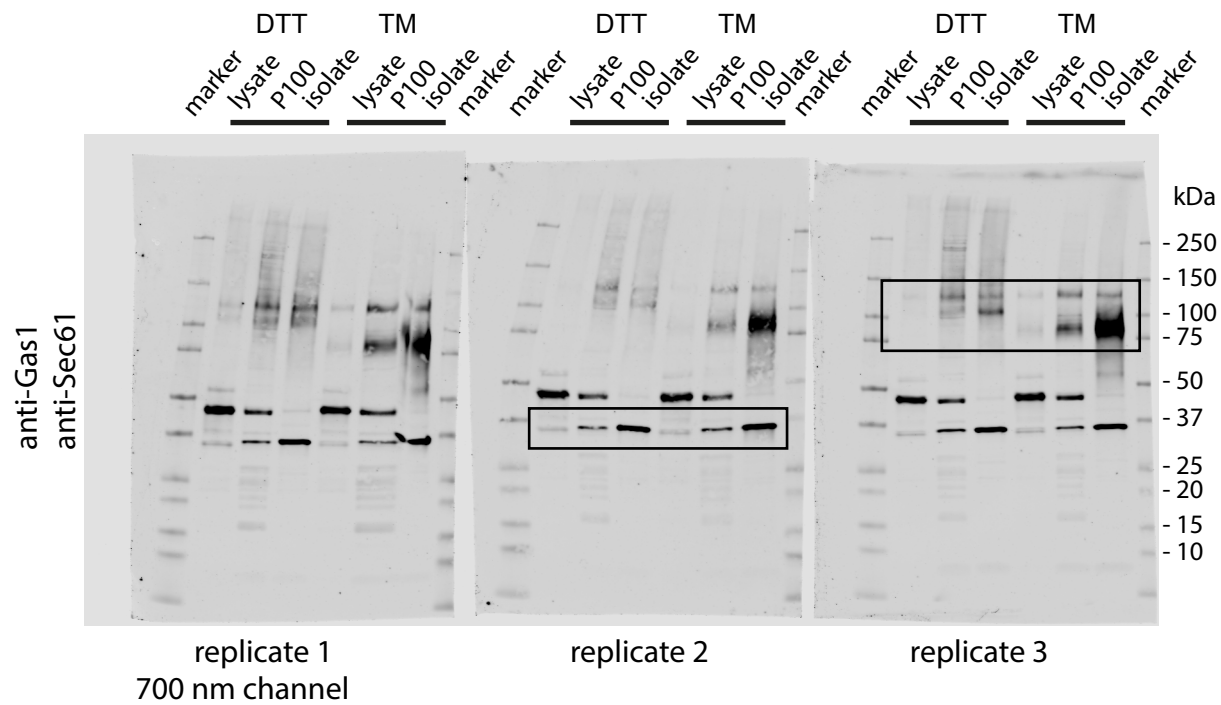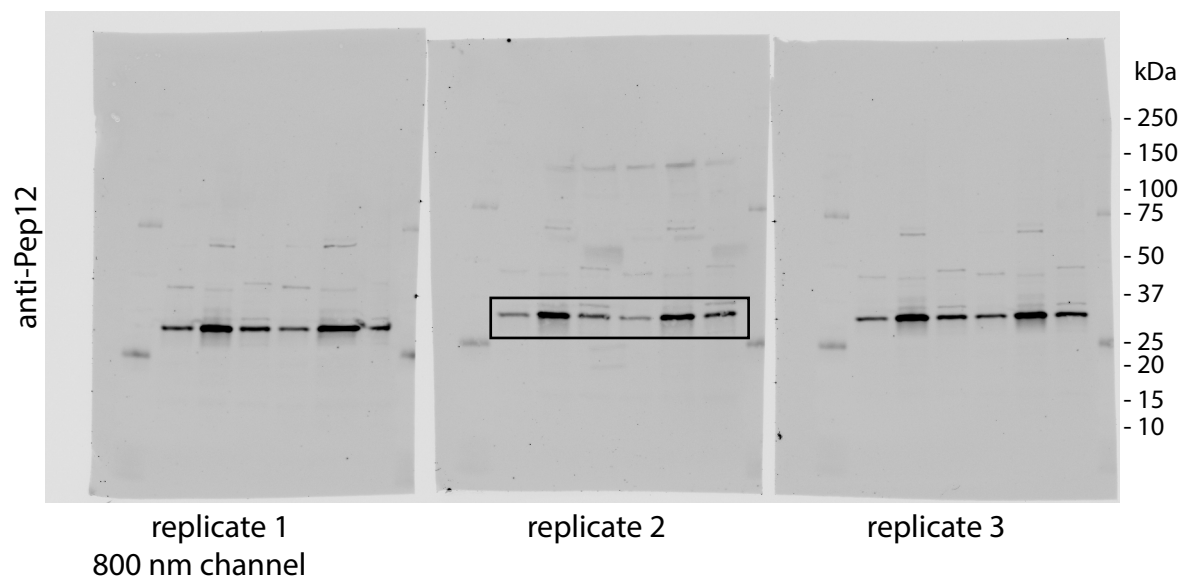

merge

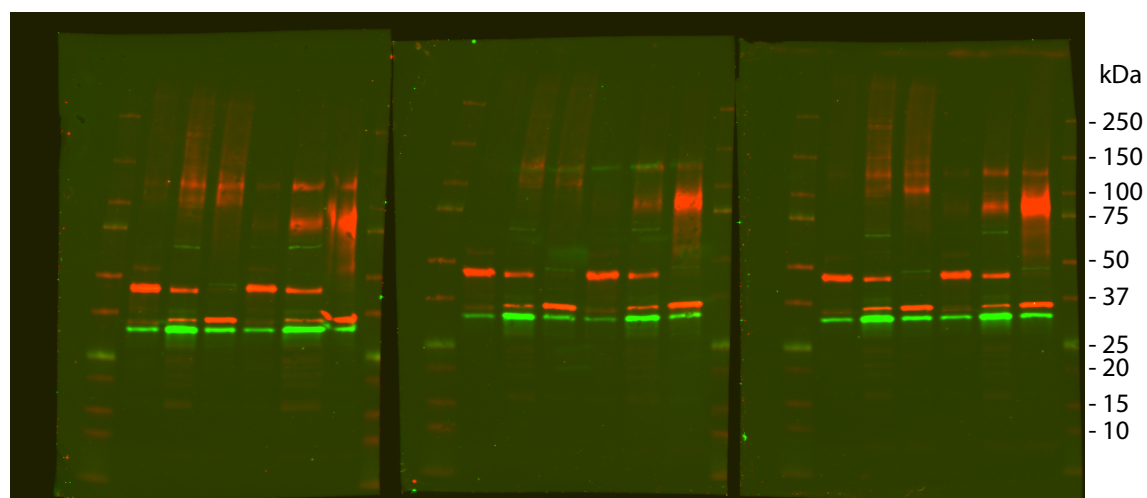

700 nm channel

800 nm channel

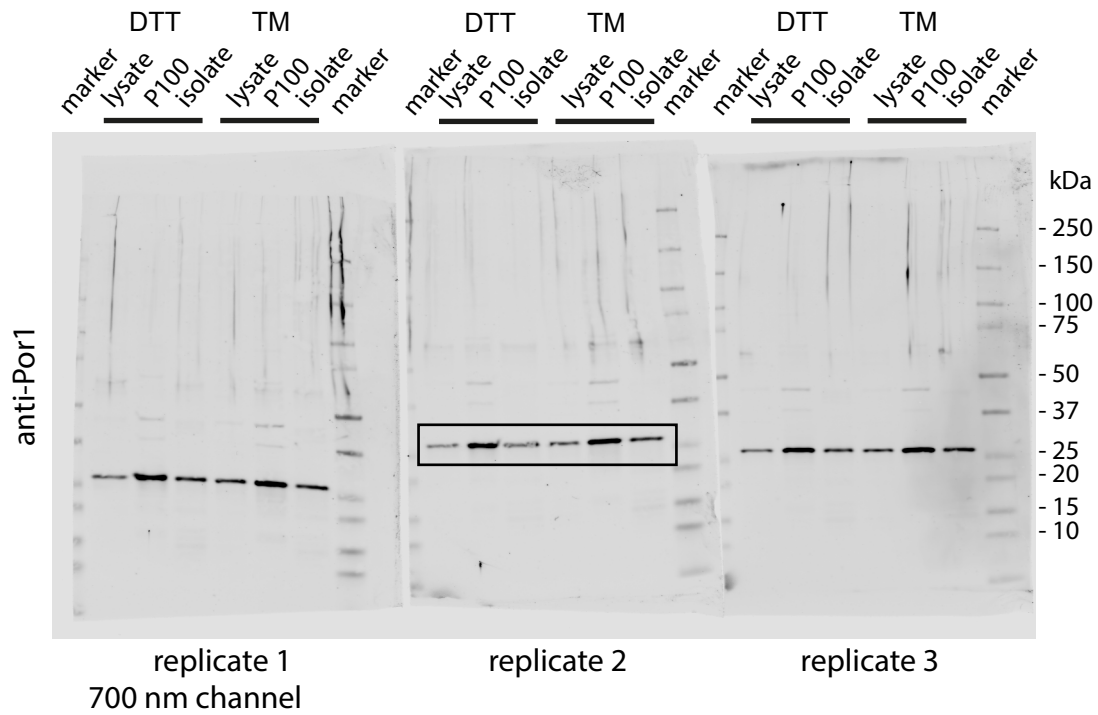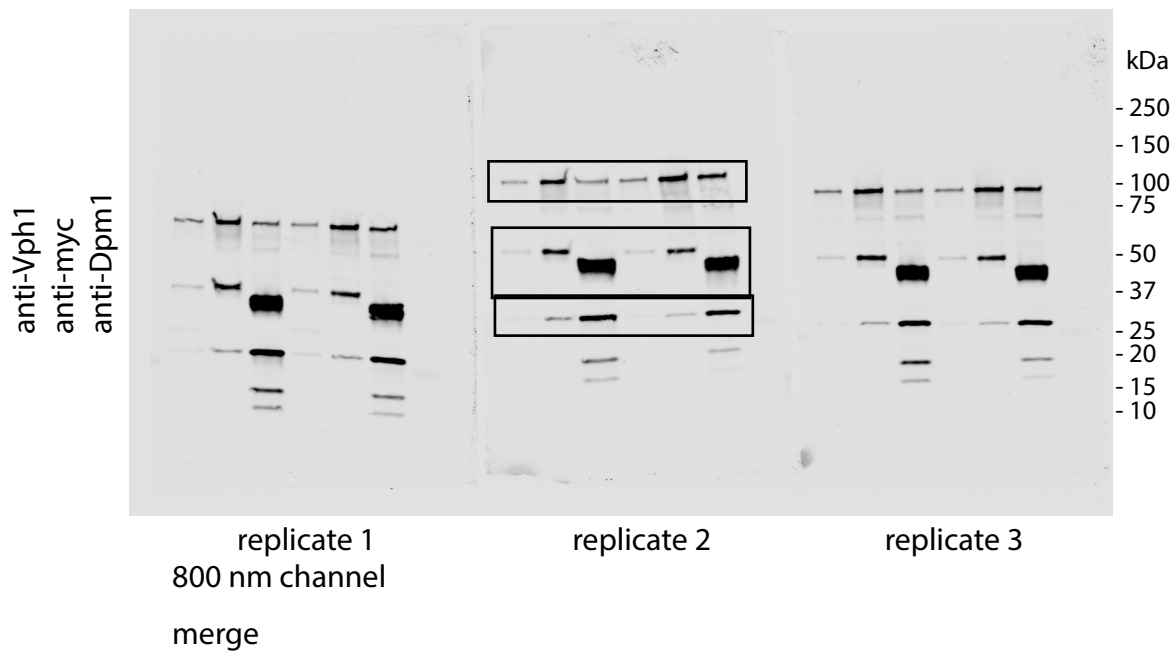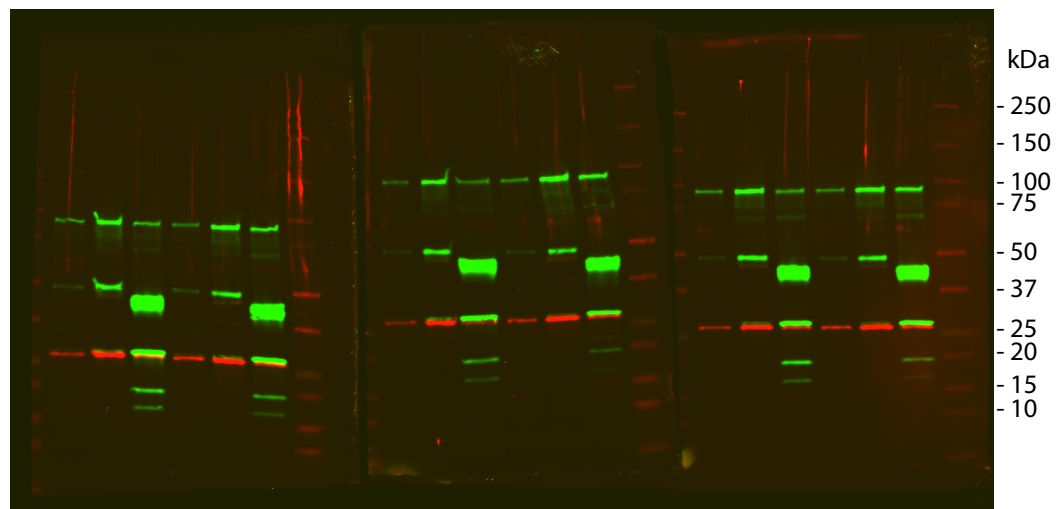

700 nm channel

800 nm channel

Supplement: Supplementary file 8 — EV and Appendix Figure Source Data [file 44318_2024_63_MOESM8_ESM.zip › Figure EV5A_immunoblots.pdf]
