# Supplementary material for: MemPrep, a new technology for isolating organellar membranes provides fingerprints of lipid bilayer stress
Source: EMBO J. 2024 Mar 15;43(8):13. doi: 10.1038/s44318-024-00063-y (PMC11021466; doi:10.1038/s44318-024-00063-y)
Supplement: Supplementary file 7 — Source Data Fig. 6 [file 44318_2024_63_MOESM7_ESM.zip › Figure 6A_immunoblots.pdf]

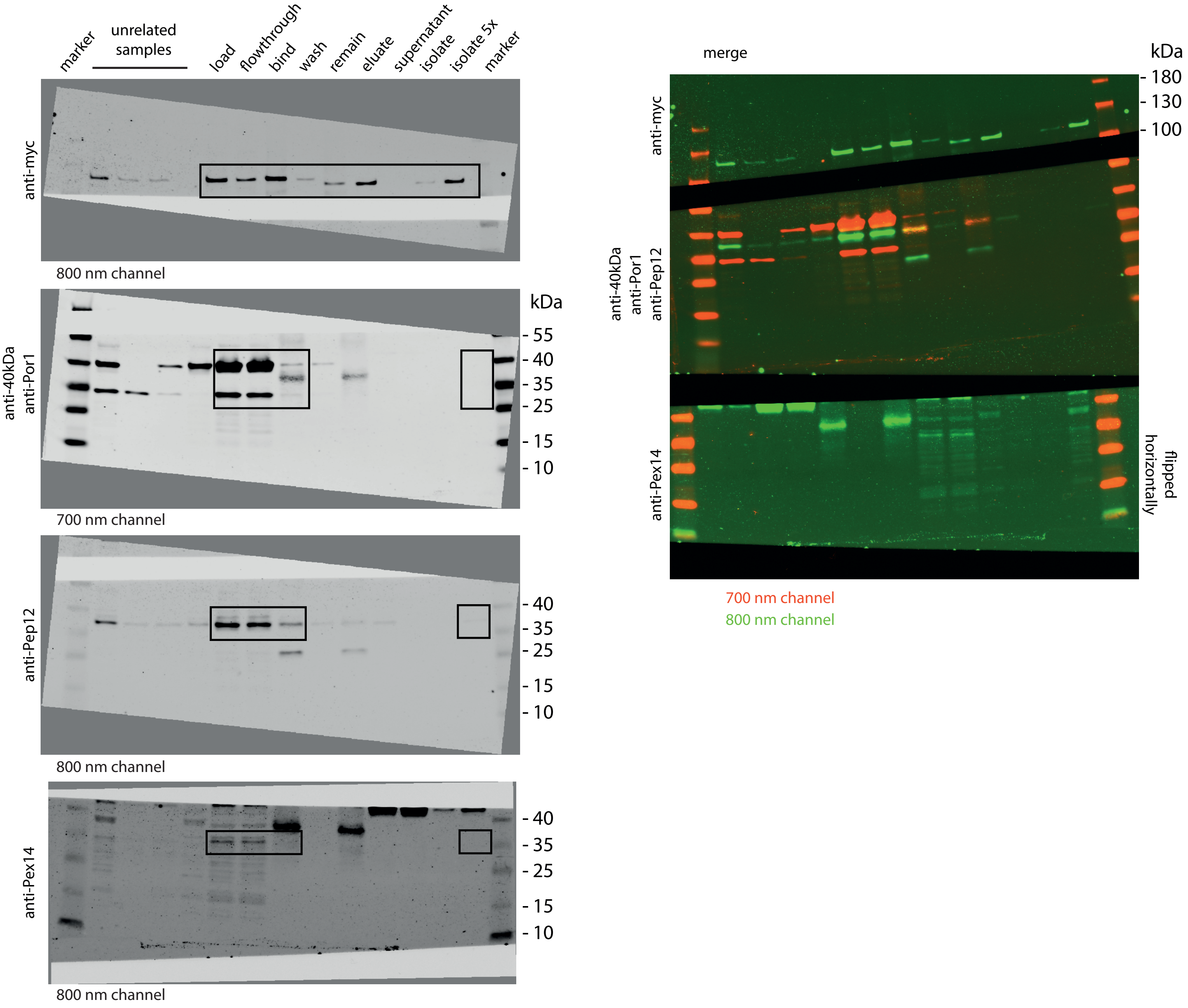

marker
unrelated samples
load
flowthrough
bind
wash
remain
eluate
supernatant
isolate
isolate 5x
marker
anti-myc
800 nm channel
anti-40kDa
anti-Por1
kDa
- 55
- 40
- 35
- 25
- 15
- 10
700 nm channel
anti-Pep12
- 40
- 35
- 25
- 15
- 10
800 nm channel
anti-Pex14
- 40
- 35
- 25
- 15
- 10
800 nm channel
merge
kDa
- 180
- 130
- 100
anti-myc
anti-40kDa
anti-Por1
anti-Pep12
anti-Pex14
flipped horizontally
700 nm channel
800 nm channel

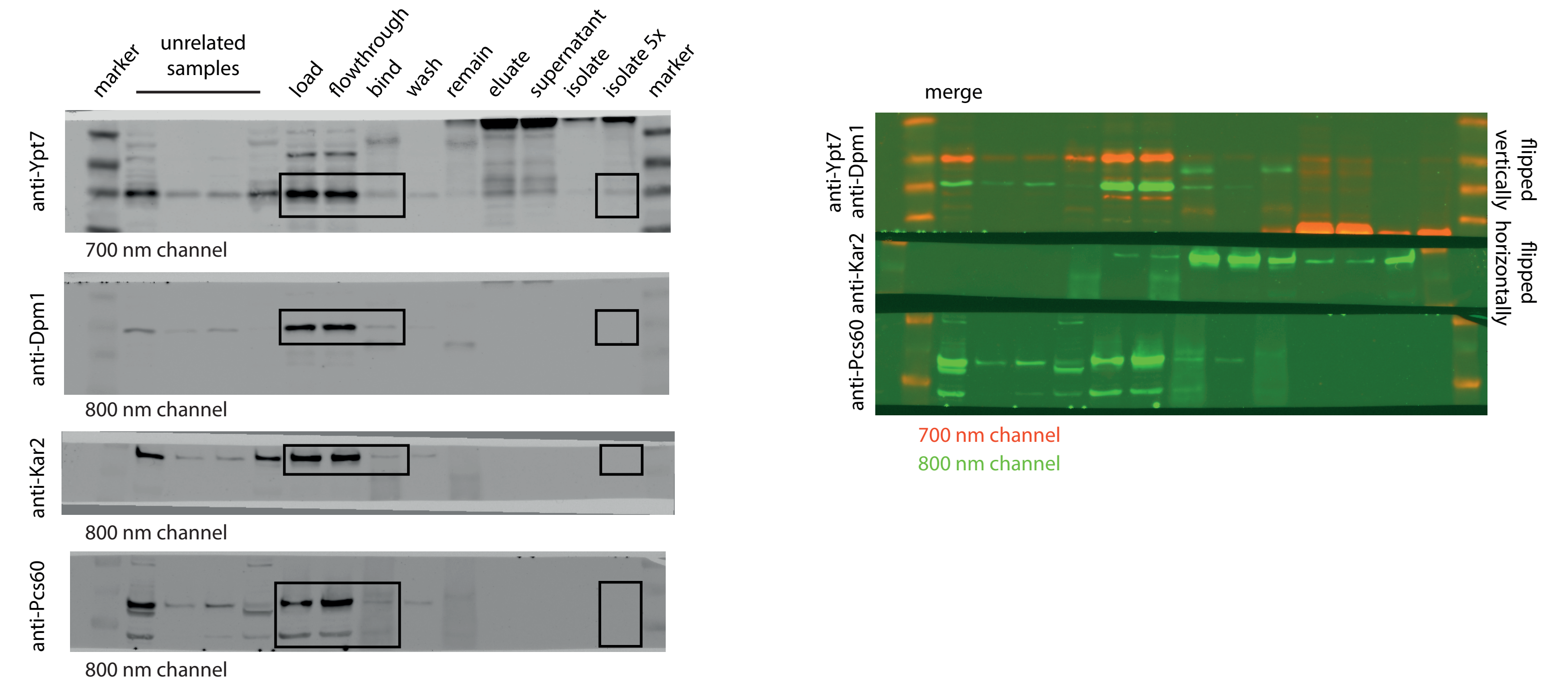

marker
unrelated samples
load
flowthrough
bind
wash
remain
eluate
supernatant
isolate
isolate 5x
marker
anti-Ypt7
700 nm channel
anti-Dpm1
800 nm channel
anti-Kar2
800 nm channel
anti-Pcs60
800 nm channel
merge
anti-Ypt7
anti-Dpm1
anti-Kar2
anti-Pcs60
flipped vertically
flipped horizontally
700 nm channel
800 nm channel

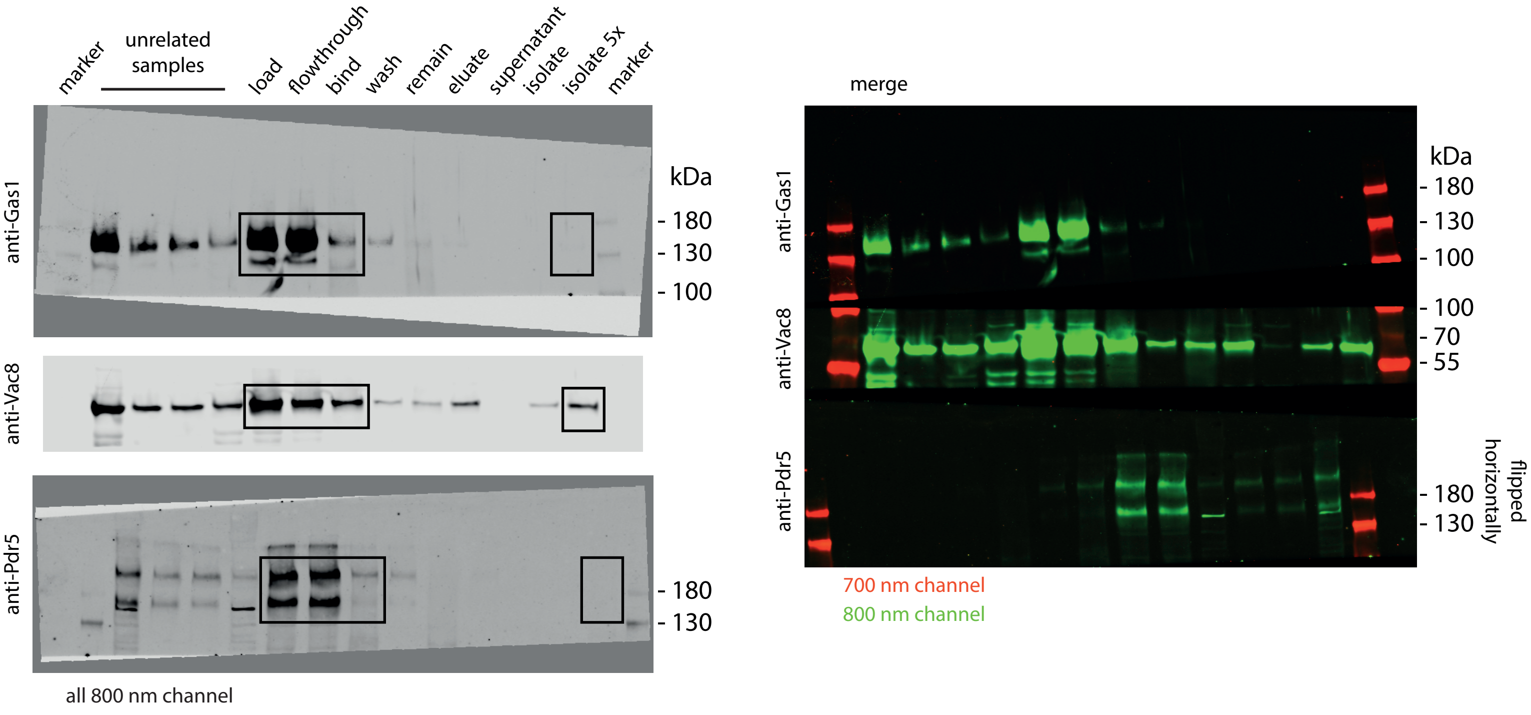

marker
unrelated samples
load
flowthrough
bind
wash
remain
eluate
supernatant
isolate
isolate 5x
marker
anti-Gas1
kDa
- 180
- 130
- 100
anti-Vac8
anti-Pdr5
- 180
- 130
all 800 nm channel
merge
anti-Gas1
kDa
- 180
- 130
- 100
anti-Vac8
- 100
- 70
- 55
anti-Pdr5
- 180
- 130
flipped horizontally
700 nm channel
800 nm channel
