# Supplementary material for: MemPrep, a new technology for isolating organellar membranes provides fingerprints of lipid bilayer stress
Source: EMBO J. 2024 Mar 15;43(8):13. doi: 10.1038/s44318-024-00063-y (PMC11021466; doi:10.1038/s44318-024-00063-y)
Supplement: Supplementary file 8 — EV and Appendix Figure Source Data [file 44318_2024_63_MOESM8_ESM.zip › Appendix Figure S1C_immunoblots.pdf]

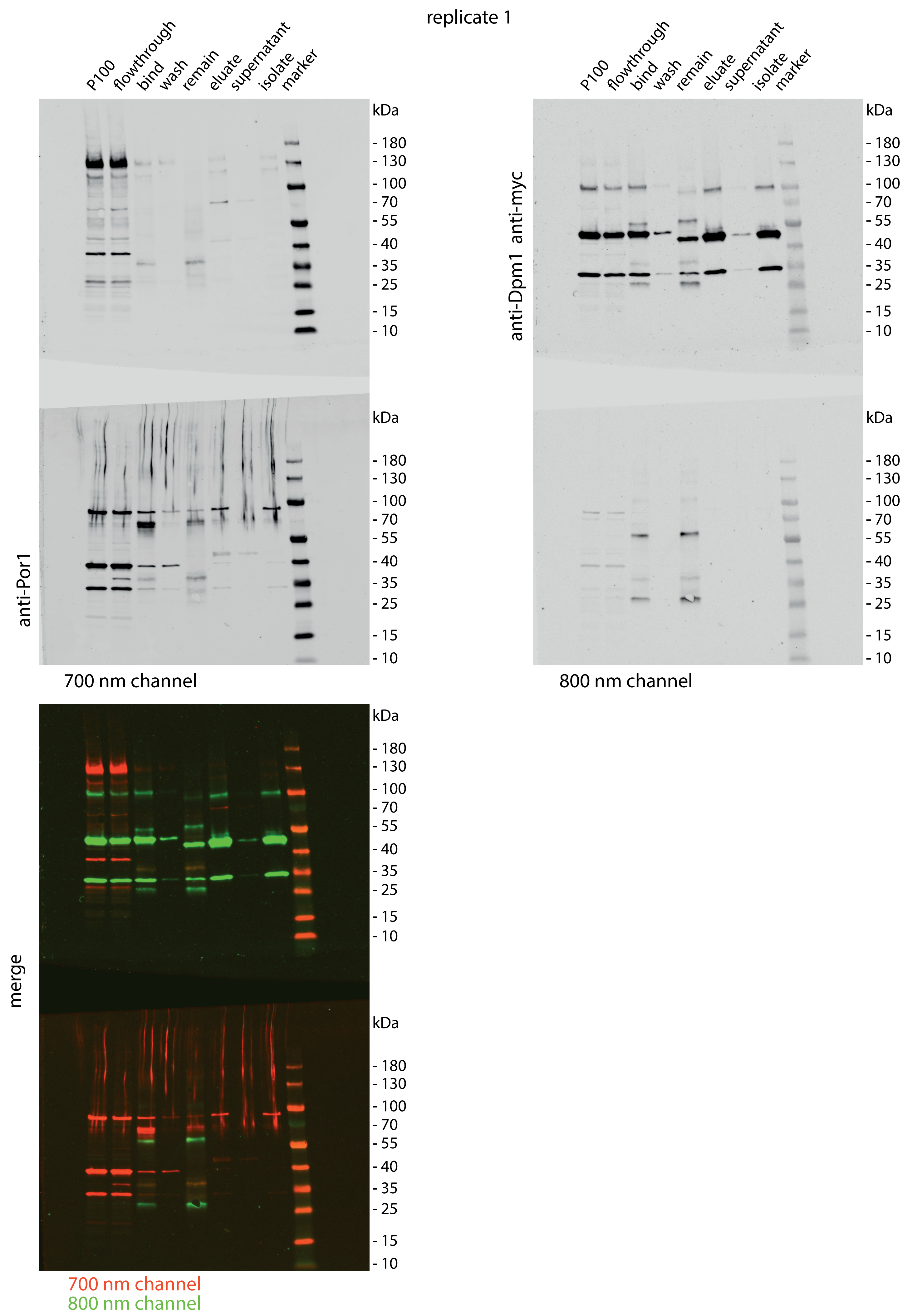

replicate 1
P100
flowthrough
bind
wash
remain
eluate
supernatant
isolate
marker
kDa
- 180
- 130
- 100
- 70
- 55
- 40
- 35
- 25
- 15
- 10
anti-myc
anti-Dpm1
anti-Por1
700 nm channel
800 nm channel
merge
700 nm channel
800 nm channel

replicate 2

same immunoblots as in Figure 2D

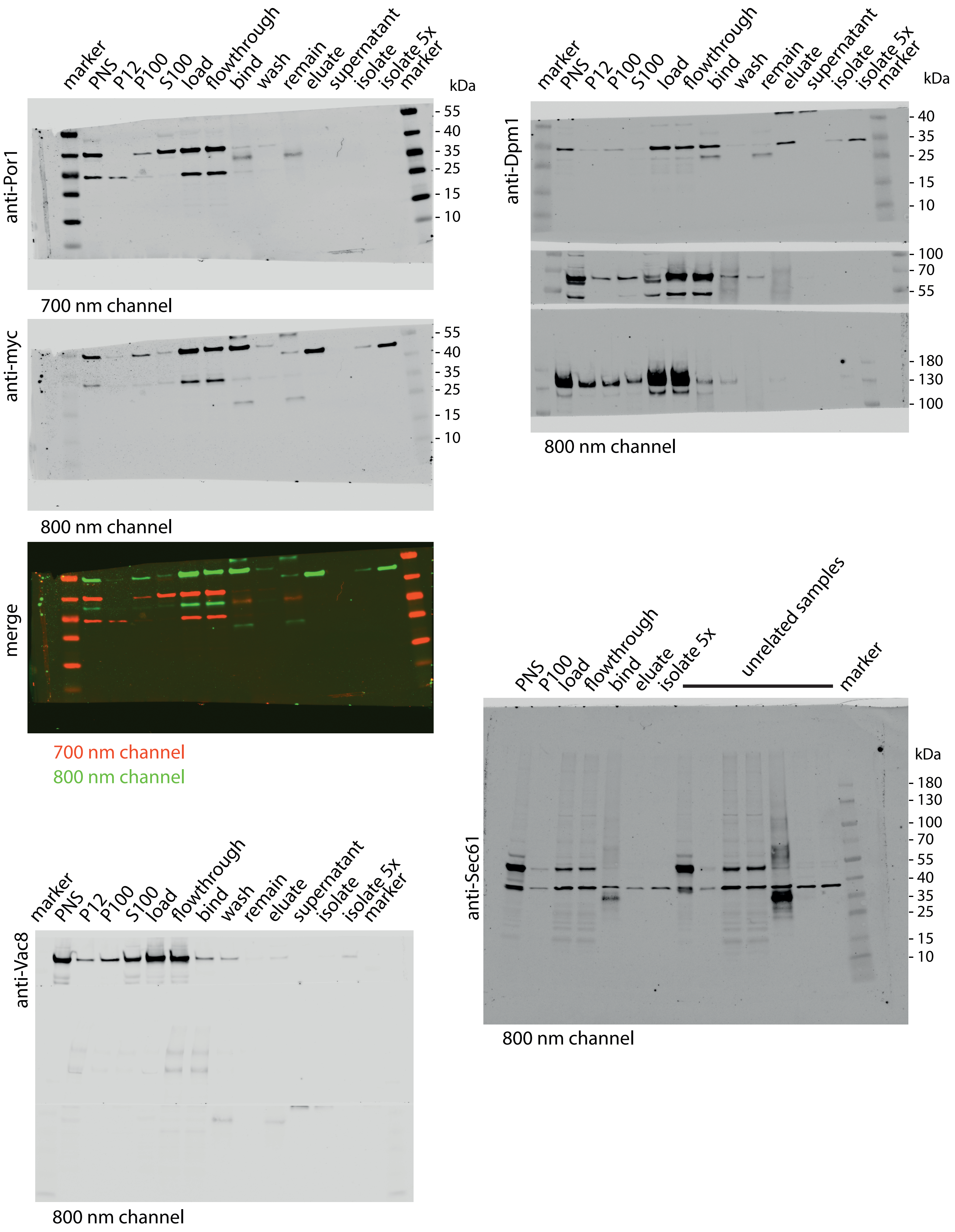

replicate 3

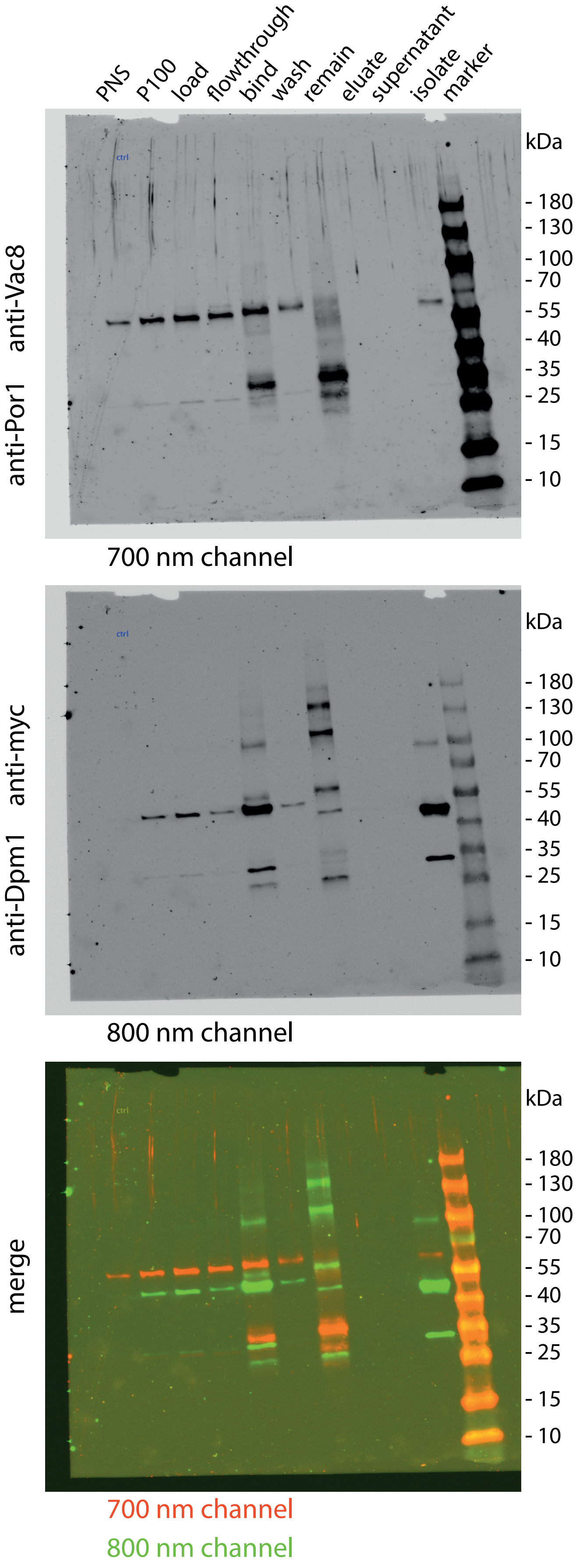

PNS
P100
load
flowthrough
bind
wash
remain
eluate
supernatant
isolate
marker
anti-Vac8
anti-Por1
kDa
- 180
- 130
- 100
- 70
- 55
- 40
- 35
- 25
- 15
- 10
700 nm channel
anti-myc
anti-Dpm1
kDa
- 180
- 130
- 100
- 70
- 55
- 40
- 35
- 25
- 15
- 10
800 nm channel
merge
kDa
- 180
- 130
- 100
- 70
- 55
- 40
- 35
- 25
- 15
- 10
700 nm channel
800 nm channel
