# Supplementary material for: MemPrep, a new technology for isolating organellar membranes provides fingerprints of lipid bilayer stress
Source: EMBO J. 2024 Mar 15;43(8):13. doi: 10.1038/s44318-024-00063-y (PMC11021466; doi:10.1038/s44318-024-00063-y)
Supplement: Supplementary file 10 — Expanded View Figures [file 44318_2024_63_MOESM10_ESM.pdf]

Expanded View Figures

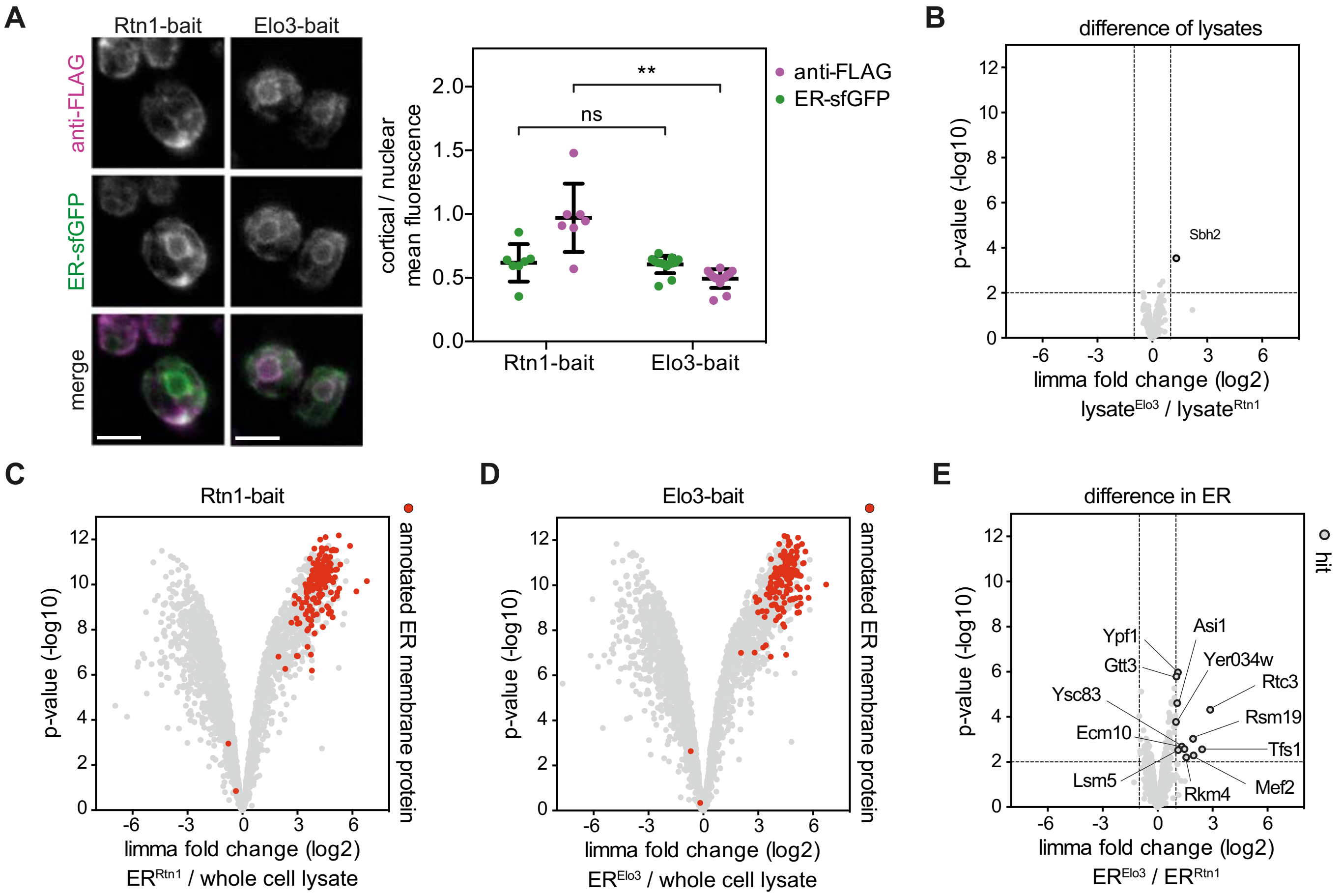

Figure EV1. ER MemPrep via two different bait proteins.

(**A**) Immunofluorescence showing the localization of two different ER membrane bait proteins (Rtn1-bait, Elo3-bait) relative to the ER-luminal marker ER-sfGFP-HDEL. Scale bar indicates 5 µm. Quantification of fluorescence distribution. Cell and nuclear areas were chosen manually. Cortical area was defined as total cellular area minus nuclear area. The ER-luminal marker ER-sfGFP-HDEL shows the same cortical-to-nuclear distribution in both bait strains. Rtn1-bait has a stronger preference for the cortical ER, compared to Elo3-bait. $n = 7$ cells for Rtn1-bait, $n = 14$ cells for Elo3-bait Data from individual cells are represented as data points yielding the average ± SD. $^{ns}P > 0.05$, $^{**}P \leq 0.01$ (unpaired parametric *t* test with Welch's correction). (**B**) Limma analysis of TMT-labeling proteomics reveals that the proteome of Rtn1-bait and Elo3-bait whole-cell lysates is identical except for a single outlier (Sbh2) ($n = 3$ biological replicates). (**C**) To increase the proteomics coverage for membrane proteins, P100 membranes were carbonate-washed before performing immunoisolation. MemPrep via Rtn1-bait enriches ER membrane proteins in the isolate (ER^{Rtn1}) ($n = 3$ biological replicates). (**D**) ER membrane proteins are enriched to the same extent by MemPrep via the bait protein Elo3-bait ($n = 3$ biological replicates). (**E**) MemPrep via Rtn1-bait and Elo3-bait yields almost identical sample composition with only 12 proteins that are enriched in the Elo3-bait derived ER. Data information: Data in (**B–E**) are presented as the mean from $n = 3$ biological replicates. A moderated t-test limma to test for differential enrichment was used. *P* values were corrected for multiple testing with the method from Benjamini and Hochberg. Source data for this figure are available online.

© The Author(s)

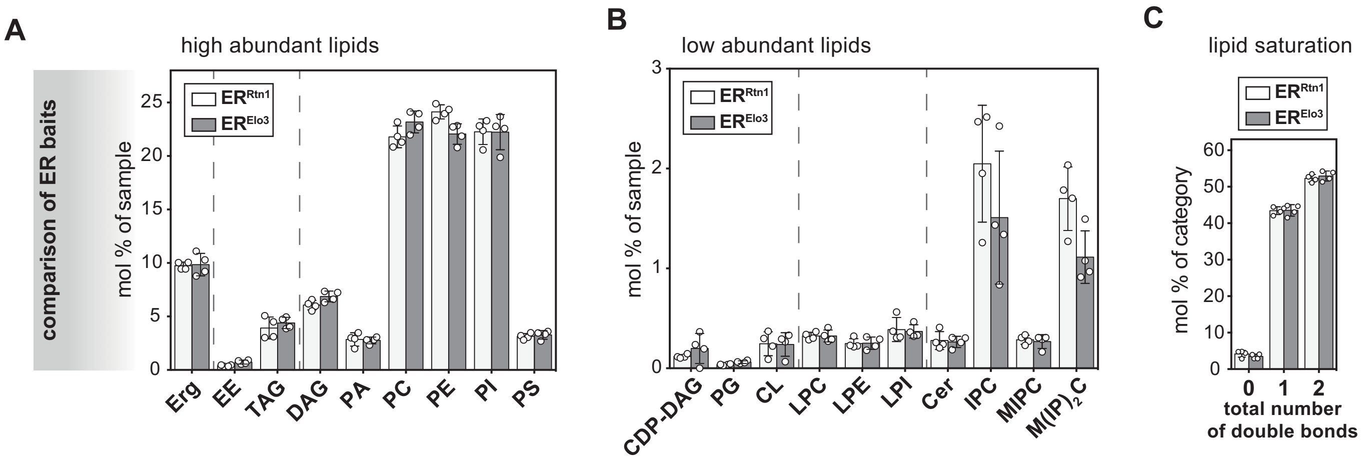

Figure EV2. The lipidome of Rtn1-bait and Elo3-bait derived ER membranes is identical.

Quantitative lipidomics of ER membranes derived via two different bait proteins. (**A**) Distribution of lipid classes with high abundance. Erg ergosterol, EE ergosteryl ester, TAG triacylglycerol, DAG diacylglycerol, PA phosphatidic acid, PC phosphatidylcholine, PE phosphatidylethanolamine, PI phosphatidylinositol, PS phosphatidylserine ($n = 4$ biological replicates). (**B**) Distribution of lipid classes with low abundance. CDP-DAG cytidine diphosphate diacylglycerol, PG phosphatidylglycerol, CL cardiolipin, LPC lyso-phosphatidylcholine, LPE lyso-phosphatidylethanolamine, LPI lyso-phosphatidylinositol, Cer ceramide, IPC inositolphosphorylceramide, MIPC mannosyl-IPC, $M(IP)_2C$ mannosyl-di-IPC ($n = 4$ biological replicates). (**C**) Total number of double bonds in membrane glycerolipids, except for CL, (i.e., CDP-DAG, DAG, PA, PC, PE, PG, PI, PS) as mol% of this category. Lipid data of Rtn1-bait derived membranes are identical with the data presented in Fig. 2A–C. Data information: In (**A**–**C**), data from $n = 4$ biological replicates are presented as individual data points and as mean ± SD. All differences of ER^{Rtn1} versus ER^{Elo3} were nonsignificant with $P > 0.05$ (multiple t tests, corrected for multiple comparisons using the method of Benjamini, Krieger and Yekutieli, with $Q = 1\%$, without assuming consistent SD). Nonsignificant comparisons are not highlighted. Source data for this figure are available online.

 © The Author(s)

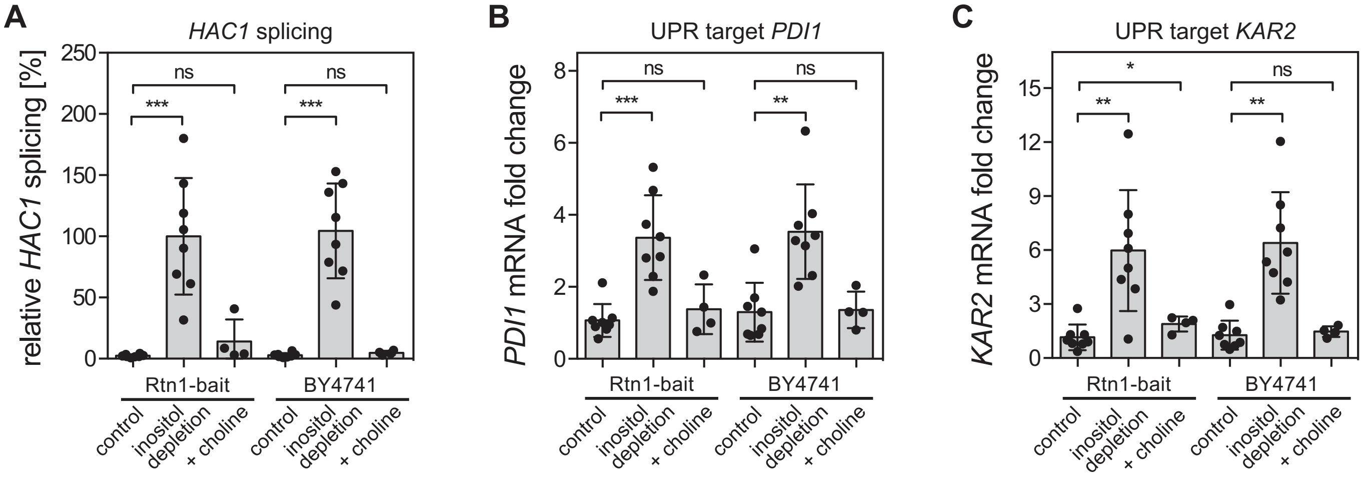

Figure EV3. Activation of the UPR by lipid bilayer stress.

SCD$_{complete}$ medium was inoculated with Rtn1-bait cells to an OD$_{600}$ of 0.003 from an overnight pre-culture and grown to an OD$_{600}$ of 1.2. Cells were washed with inositol-free medium and then cultivated for an additional 2 h in either inositol-free (inositol depletion) or SCD$_{complete}$ medium (control) starting with an OD$_{600}$ of 0.6. Another perturbation of lipid metabolism was achieved by addition of choline. For '+choline' conditions, SCD$_{complete}$ medium was inoculated to an OD$_{600}$ of 0.1 using stationary overnight cultures. Cells were then cultivated to an OD$_{600}$ of 1.0 in the presence of 2 mM choline. (**A**) UPR activation was measured by determining the levels of spliced *HAC1* mRNA. Data for relative *HAC1* splicing was normalized to the inositol depletion Rtn1-bait condition ($n = 8$ biological replicates based on two technical replicates for Rtn1-bait control, Rtn1-bait inositol depletion, BY4741 control and BY4741 inositol depletion, but $n = 4$ biological replicates based on two technical replicates for Rtn1-bait + choline and BY4741 + choline). (**B**) mRNA upregulation of the downstream UPR target gene *PDI*. *PDI* mRNA fold change was calculated as $2^{-\Delta\Delta Ct}$ and normalized to Rtn1-bait control condition ($n = 8$ biological replicates based on two technical replicates for the Rtn1-bait control, Rtn1-bait inositol depletion, BY4741 control, and BY4741 inositol depletion, but $n = 4$ biological replicates based on two technical replicates for Rtn1-bait + choline and BY4741 + choline). (**C**) Upregulation of mRNA of the downstream UPR target gene *KAR2* calculated as $2^{-\Delta\Delta Ct}$ and normalized to Rtn1-bait control condition ($n = 8$ biological replicates based each on two technical replicates for Rtn1-bait control, Rtn1-bait inositol depletion, BY4741 control, and BY4741 inositol depletion, but $n = 4$ biological replicates based on two technical replicates for Rtn1-bait + choline and BY4741 + choline). Data information: All data from biological replicates are presented in (**A–C**) as individual data points with the mean ± SD. $^{ns}P > 0.05$, $^{*}P \leq 0.05$, $^{**}P \leq 0.01$, $^{***}P \leq 0.001$ (unpaired parametric *t* test with Welch's correction). Nonsignificant comparisons are not highlighted. Source data for this figure are available online.

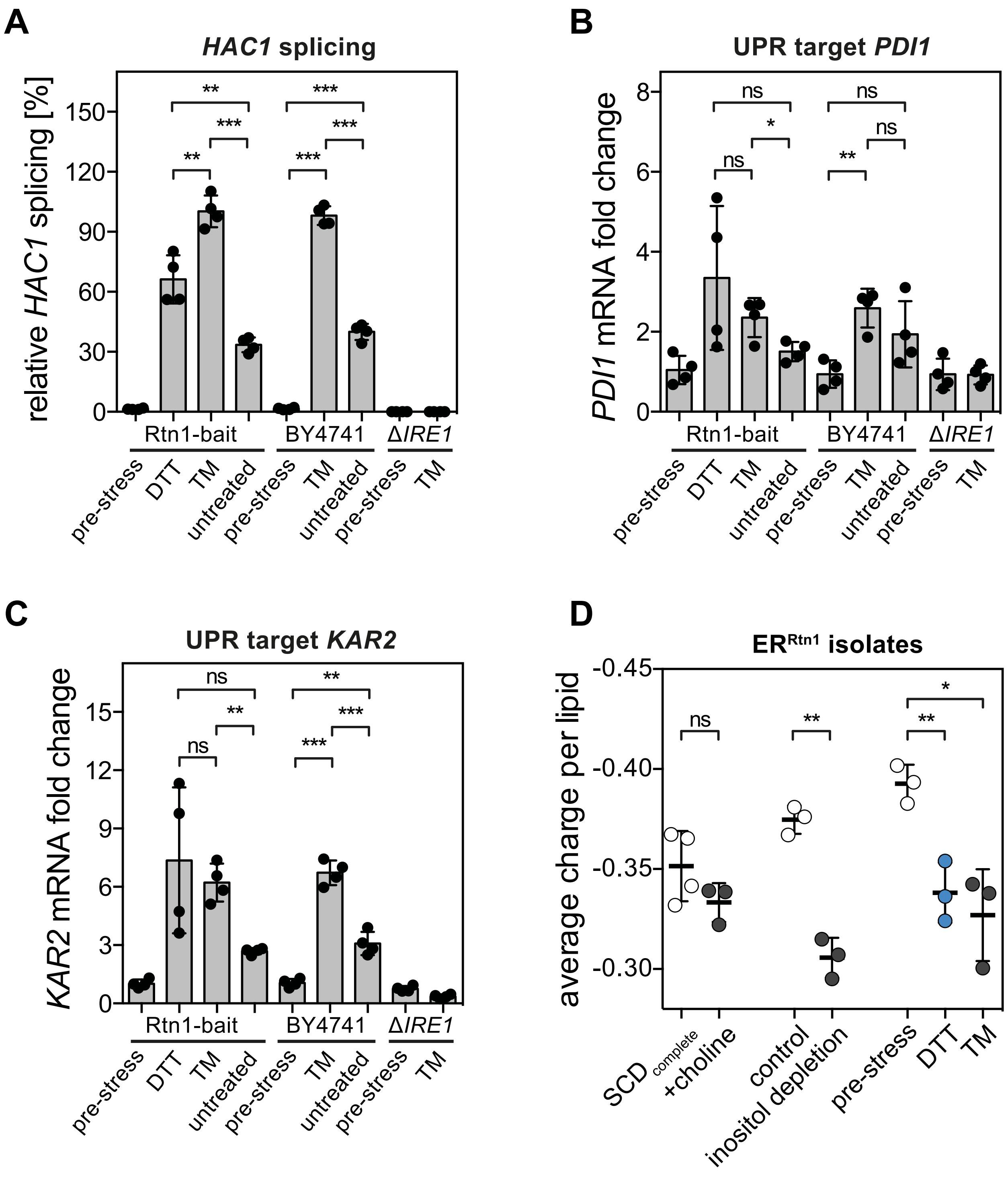

Figure EV4. Activation of the UPR upon prolonged proteotoxic stress.

Cells were grown as described above. UPR activation was measured by determining the levels of (**A**) spliced *HAC1* mRNA and the mRNA of the downstream UPR target gene ($n = 4$ biological replicates based on two technical replicates) (**B**) *PDI* ($n = 4$ biological replicates based on two technical replicates) and (**C**) *KAR2* before and after 4 h of DTT or TM treatment ($n = 4$ biological replicates based on two technical replicates). Data for relative *HAC1* splicing was normalized to the TM-treated Rtn1-bait condition. *PDI* and *KAR2* mRNA fold changes were calculated as $2^{-\Delta\Delta Ct}$ and normalized to Rtn1-bait pre-stress. (**D**) Calculation of the average charge per lipid from ER lipidomics data shown in Appendix Fig. S3A,B ($SCD_{complete}$, +choline), Fig. 3B,C (control, inositol depletion), and Fig. 4D,E (pre-stress, DTT, TM). Conditions with active UPR show reduced negative lipid charges compared to their respective controls. Net charges of the lipid classes were considered as follows: Erg 0, EE 0, TAG 0, DAG 0, PA -1, PC 0, PE 0, PI -1, PS -1, CDP-DAG -2, PG -1, CL -2, Cer 0, IPC -1, MIPC -1, $M(IP)_2C$-2 ($n = 4$ biological replicates for $SCD_{complete}$ and $n = 3$ biological replicates for all other conditions). Data information: All data are presented as individual data points and the mean ± SD. $^{ns}P > 0.05$, $^{*}P \leq 0.05$, $^{**}P \leq 0.01$, $^{***}P \leq 0.001$ (unpaired parametric *t* test with Welch's correction). Source data for this figure are available online.

© The Author(s)

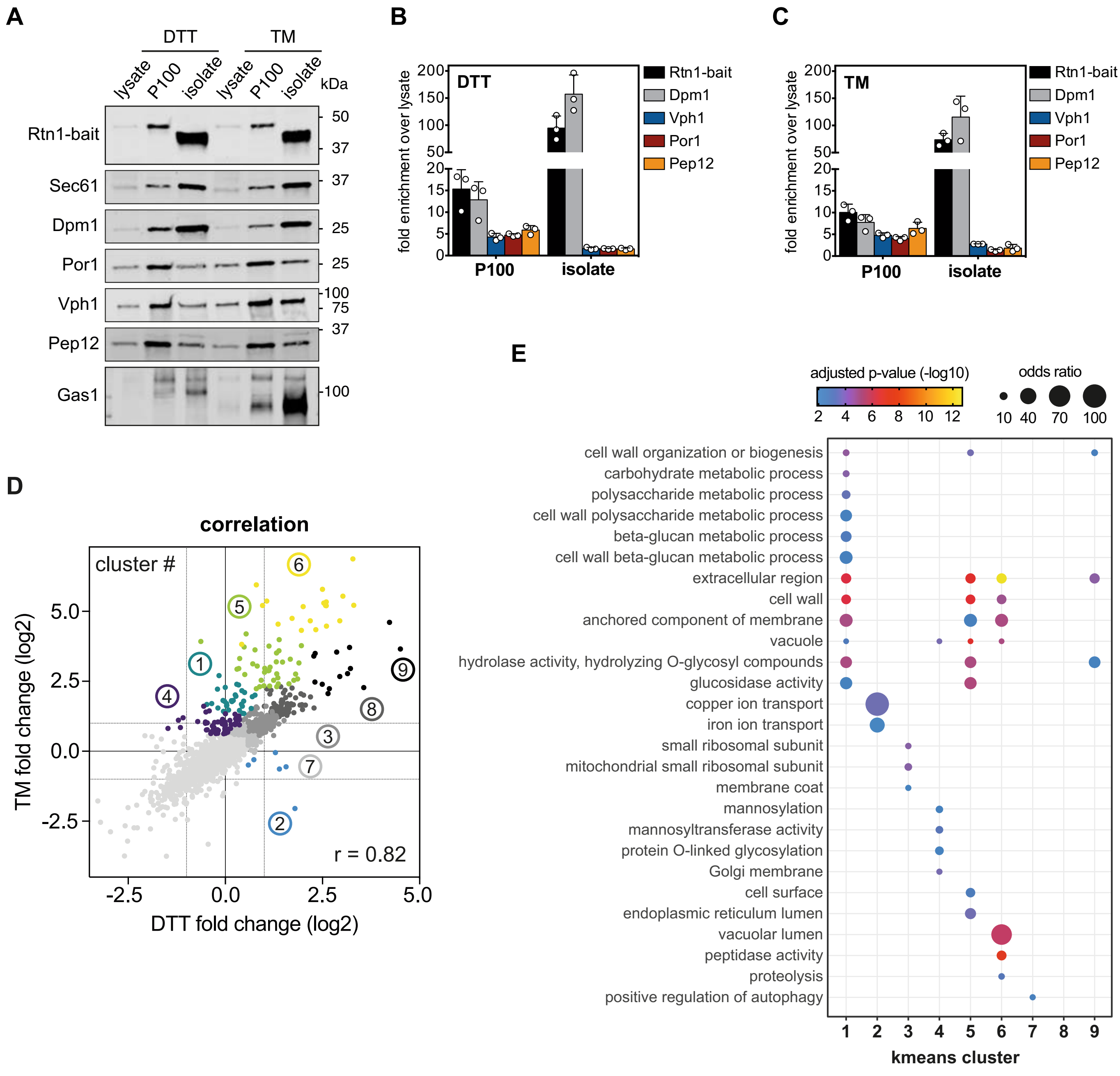

Figure EV5. Enrichment of stressed ER membranes by MemPrep.

(**A**) Immunoblot analysis of the indicated organellar markers in whole-cell lysates (lysate), crude membranes (P100), and MemPrep isolates (isolate). ER membranes were immuno-isolated via the Rtn1-bait protein. Sec61 and Dpm1 are prototypical ER membrane markers. Por1 is a marker for the outer mitochondrial membrane, Vph1 is a vacuolar marker. Pep12 marks endosomes and Gas1 serves as plasma membrane marker. 1 µg total protein loaded per lane. (**B**) Quantification of the organelle markers Dpm1, Vph1, Por1, Pep12 and the Rtn1-bait protein from three immunoblots of independent replicate ER MemPreps after prolonged proteotoxic stress induced by DTT ($n = 3$ biological replicates). (**C**) Quantification of three immunoblots from independent replicate ER MemPreps after prolonged proteotoxic stress induced by TM. Error bars indicate standard deviations ($n = 3$ biological replicates). (**D**) Correlation of DTT- and TM-induced fold changes, after Limma analysis, over pre-stress with a Pearson correlation coefficient $r = 0.82$. K-means clusters are indicated by colored groups and their respective cluster number ($n = 3$ biological replicates). (**E**) Gene ontology term enrichments in K-means clusters ($n = 3$ biological replicates). Data information: Data in (**B**, **C**), data from three biological replicates are presented as individual data points and as the mean ± SD. Data in (**E**) from $n = 3$ biological replicates are presented as the mean. *P* values were derived from a Fisher-test and corrected for multiple testing with the method of Benjamini and Hochberg. Source data for this figure are available online.

© The Author(s)
